# Sparking angiogenesis by carbon monoxide-rich gold nanoparticles obtained by pulsed laser driven CO_2_ reduction reaction

**DOI:** 10.1186/s12951-025-03680-9

**Published:** 2025-08-26

**Authors:** Anastasia Chillà, Cecilia Anceschi, Francesca Scavone, Serena Martinelli, Jessica Ruzzolini, Elena Frediani, Francesca Margheri, Tahir Tahir, Guilherme C. Concas, Mariana Gisbert, Marco Cremona, Fernando Freire, Ricardo Q. Aucélio, Tatiana Saint Pierre, André L. Rossi, Mirko Severi, Rita Traversi, Daniele Bani, Daniele Guasti, Nicola Daldosso, Mario Del Rosso, Gabriella Fibbi, Celso SantAnna, Tommaso Del Rosso, Anna Laurenzana

**Affiliations:** 1 Department of Experimental and Clinical Biomedical Sciences, University of Florence, Viale Morgagni 50, Florence, 50134 Italy; 2 Department of Experimental and Clinical Medicine, University of Florence Florence, Viale Pieraccini 6, 50134 Florence, Italy; 3 Department of Physics, Pontifical Catholic University of Rio de Janeiro, Rua Marquês de São Vicente 225, Gávea, Rio de Janeiro, 22451-900 Brazil; 4 Department of Chemistry, Pontifical Catholic University of Rio de Janeiro, Rua Marquês de São Vicente 225, Gávea, Rio de Janeiro, 22451- 900 Brazil; 5 Centro Brasileiro de Pesquisas Físicas (CBPF), R. Dr. Xavier Sigaud 150, Urca, Rio de Janeiro, 22290-180 Brazil; 6 Department of Chemistry, Ugo Schiff University of Florence Sesto, Fiorentino, 50019 Italy; 7 Department of Engineering for Innovation Medicine, University of Verona, Strada le Grazie 15, Verona, 37134 Italy; 8 Duque de Caxias (RJ), National Institute of Metrology Quality and Technology (Inmetro), Av. Nossa Senhor das Graças 50, 25250-020 Rio de Janeiro, Brazil

**Keywords:** Carbon monoxide, Gold nanoparticles, Pulsed laser ablation in water, Endothelial colony forming cells, Capillary morphogenesis, Histones acetylation; matrigel sponges; angiogenesis

## Abstract

Endothelial tissue regeneration is a major challenge in the context of vascular disorders and tissue repair. One of the most recent and promising therapies for endothelial tissue disorders is the administration of carbon monoxide (CO) by direct injection or release by CO-releasing molecules (CORMs). Despite the great potential of CORMs, light instability and cytotoxicity associated with the heavy metal core are still major drawbacks that inhibit clinical application. Recently, we have shown the possibility to synthesize carbon monoxide rich gold nanoparticles (CO-rich AuNPs) by the pulsed laser driven CO_2_ reduction reaction in water. In this work, we investigate the potential of this unique metal-organic complex as a therapeutic approach to promote endothelial tissue regeneration, by performing a comparative analysis between the CO releasing potential of CO-rich AuNPs and a well-known CO-releasing molecule, specifically CORM-2. Through a combination of in vitro and in vivo experiments, we elucidated the mechanisms by which the laser synthesized ligand-free or CO-rich AuNPs inhibit or enhance, respectively, the endothelial cell migration and angiogenesis. Notably, the biocompatible CO-rich AuNPs not only boost these cellular processes but also play a pivotal role in triggering histone acetylation, shedding light on an additional regulatory pathway.

**Graphical abstract:**

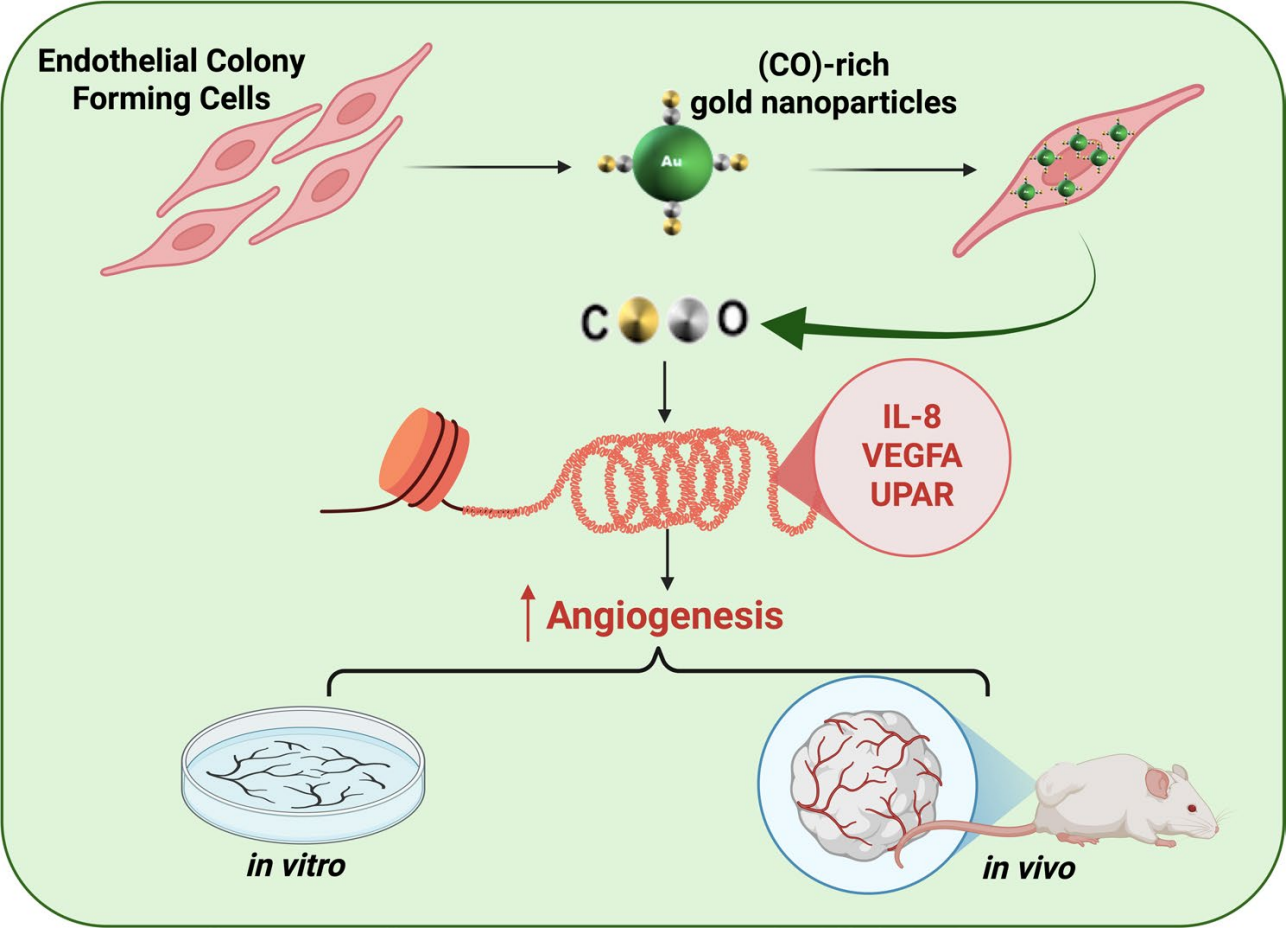

**Supplementary Information:**

The online version contains supplementary material available at 10.1186/s12951-025-03680-9.

## Introduction

Gold nanoparticles (AuNPs) have been intensively studied in the last decades, due to a variety of attractive physic-chemical properties, such as high oxidation stability, high biocompatibility, versatile surface functionalization and localized plasmonic resonance [1–4]. The combination of these properties is the key to their wide application in the field of theranostics, where AuNPs play a fundamental role as local heat emitters, photo-acoustic transducers, optical nanoantennas for the enhancement of spectroscopic signals in bioimaging, and drug nano-carriers for regenerative medicine [4–9].

Among the different strategies described in literature for the preparation of gold nanostructures, chemical reduction [10], biological approach [11] and pulsed laser ablation in liquid (PLAL) [12], are probably the most widely used techniques for the synthesis of gold nanoparticles. While the presence of by-products is inherent in the wet chemical synthesis of AuNPs, the traditional literature on PLAL introduced the concept of ligand-free gold nanoparticles [13], due to the absence of by-products and ligand agents on the metal surface.

Contrary to the thesis reported above, we have recently discovered that the CO_2_ reduction reaction (CO_2_RR) [14] is an intrinsic process of PLAL at the air-water interface, where the products of the reaction become macroscopically observable when a larger amount of aqueous CO_2_ is introduced into a basic water environment. The pulsed laser driven CO_2_RR using gold targets, leads to the formation of various metal-organic nanostructures, in particular of organometallic nanocomposites (OMNCs) composed of small gold nanoclusters and carboxylic acid salts (di-hydrated sodium formate, sodium acetate and sodium lactate), and carbon monoxide-rich gold nanoparticles (CO-rich AuNPs). The latter are surprisingly produced also when PLAL is performed in pure water in equilibrium with air [15].

The importance in the synthesis of transition metal carbonyl complexes in medicine, is due to the fundamental role of carbon monoxide as mediator in various processes underlying inflammatory and vascular diseases [16–18]. In addition to the best-known vascular diseases, characterized by vessel occlusion with often fatal consequences, human vascular pathology includes diseases characterized by progressive small vessels involution. Such diseases encompass, but are not limited to, cerebral small vessel disease [19], Systemic sclerosis (SSc) [20], chronic obstructive pulmonary disease (COPD) [21], inflammatory bowel diseases, such as Chron disease and ulcerative colitis [22], coronary microvascular disease (CMD), often associated with increased risk of adverse effects [23]. Despite progress in pharmaceutical research and surgery technologies the treatment of these diseases remains challenging. It is obvious that using scaffolds capable of supporting good vascular growth is essentially applicable in the vascular regeneration of the skin, which is the largest organ in the human body. Since the skin is essentially organized in two dimensions, with the third dimension almost negligible compared to the other two, devices for vascular skin tissue engineering have the possibility of obtaining notable successes in localized revascularization processes, as previously discussed [24]. In consideration of the pan-organic nature of the aforementioned diseases, which often affect several organs at the same time, or a single organ in its entirety, the use of nano scaffolds comes up against severe application obstacles.

A great effort is underway to identify the best in vivo delivery strategies for nanoparticle-based therapeutics [25, 26], and the influence of different nanostructural properties in directing the cellular responses to biomaterials [27], in particular considering nanoparticles intrinsically endowed with pro- and anti-angiogenic effects independent of active targeting. A recent review by Cui et al. [28] lists the pro-angiogenic mechanisms of NPs composed of graphene oxide, zinc oxide, cerium oxide, and europium hydroxide. More recently, copper-based nanomaterials have shown strong potential: Zhang et al. developed a CuS-based hydrogel that promotes angiogenesis and M2 macrophage polarization [29], while Peng et al. reported a microneedle patch enabling sequential release of CuO₂ and VEGF for synergistic antibacterial and pro-angiogenic effects [30]. In contrast, several mechanisms have been identified in the use of chemically synthesized AuNPs as therapeutic agents to control pathological angiogenesis [28].

Moreover, the administration of mesenchymal stem cells (MSCs) via systemic injection [31] has arisen as a proposed strategy to foster vascular regeneration. Nevertheless, the emergence of potential and unforeseen risks poses a significant concern in the context of stem cell-based regenerative therapies [32].

The seminal papers by Dulak et al. [18] describing the angiogenic capabilities of CO, and Motterlini and Otterbein [33] elucidating the CO therapeutic potential, have stimulated a new line of research aimed to exploit the pro-angiogenic properties of CO. The so called CO-Releasing Molecules (CORMs) have rapidly gained space in the scientific literature as efficient and safe compounds with a biological impact on redox control, cytoprotection, modulation of the innate immune system and vasoactive response [34]. The major drawbacks of CORMs are the rapid release of the CO under physiological conditions, which prevents the proper targeting of the drug [35], the instability related to oxidation and degradation in light and atmosphere (i.e. storage and transport limitations) [36], and the cytotoxicity associated with the heavy metal core, which provokes uncontrolled reactions with the cell nucleus and membrane, increasing cellular apoptosis and necrosis [35, 37, 38]. In this scenario, the potential development of CO vectors for clinical applications seems to be limited to the possibility to synthesize coordination complexes based on metals with low cytotoxicity, such as gold.

In the present article we demonstrate the possibility to switch from an anti- to a pro-angiogenic behavior of AuNPs synthesized by PLAL, depending on the gas environment in equilibrium with the water during the synthesis. When Au-carbonyl is present on the surface of the AuNPs, a subpopulation of endothelial cell progenitors called endothelial colony-forming cells (ECFCs), following phagocytosis of the CO-rich AuNPs undergo a strong neoangiogenic push. Such AuNPs act in a CORM-like fashion and induce a CO/NO-dependent angiogenesis process, sustained by PI3K/pAKT/mTOR activation. We have validated the pro-angiogenic activity of AuNPs in vitro and in sub-dermal Matrigel sponges in nude mice, demonstrating the potential of the CO-rich AuNPs for endothelial tissue proliferation and organization.

## Results and discussion

### Dimensional and chemical characterization of the AuNPs

As reported in the Materials and Methods section, the AuNPs were synthesized by both citrate chemical reduction (AuNPs_ch_) [39] and PLAL in water at equilibrium with different gaseous environment: air (AuNPs_air_), argon (AuNPs_arg_), or a mixture of argon (99%) with CO_2_ (1%) (AuNPs_CO2_, percentages relative to the partial pressure of the mixture).

**Fig. 1:**
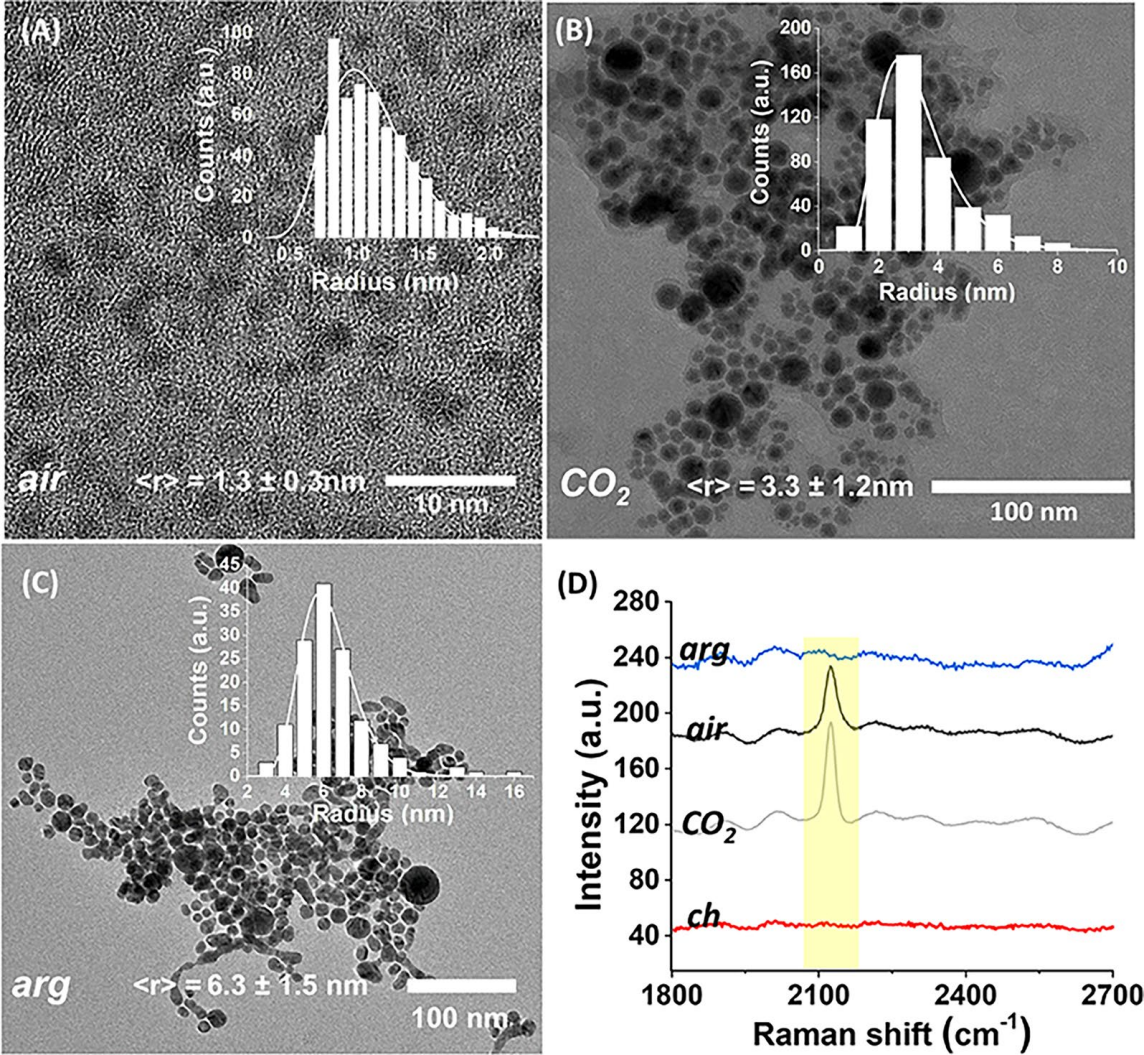
Transmission electron microscopy images of the different synthesized nanomaterials: (**A**) AuNPs_air_; (**B**) AuNPs_CO2_; (**C**) AuNPs_arg_. The insets show the experimental statistical size (radius) log-normal distribution. (**D**) SERS spectra of the AuNPs synthesized by PLAL at different gas-water interfaces, and of the citrate-stabilized AuNP (ch, red line). Only the spectral region of interest between 1800 cm^−1^ and 2700 cm^−1^ is shown for convenience. In yellow is indicated the region corresponding to the gold-carbonyl vibration. The baseline of each spectra was artificially shifted for a better visualization

In Fig. 1(A-C), are shown the transmission electron microscopy images and the log-normal statistical size distribution [40] of the AuNPs synthesized by different approaches. As previously reported, PLAL at the water-air interface by simultaneous pulses at the wavelength of 1064 nm and 532 nm, leads to ultrasmall nanoparticles with an average diameter of about ∼ 3 nm, which are in part embedded in the organic acids produced during the pulsed laser driven CO_2_ reduction reaction [15]. This particular experimental configuration for the synthesis of the AuNPs_air_ was chosen for two main reasons: (i) ultrasmall AuNPs are more efficient in passing through cellular membranes [41–43]; (ii) it allows the production of both organic and inorganic materials with potential biological effect in endothelial cells, such as Au-CO complexes together with acetic and lactic acid in free state or complexed with gold nanoclusters [15].

The AuNPs_arg_ and AuNPs_CO2_, present larger dimensions than AuNPs_air_, with an average diameter of ∼13 and ∼7 nm, respectively. Their partial agglomeration in colloidal phase, is reflected in the normalized extinction spectra of the different samples shown in Fig. S1, where the interaction between the nanoparticles leads to the broadening of the localized surface plasmon resonance [44]. The AuNPs synthesized by chemical routes (AuNPs_ch_) have been characterized morphologically and dimensionally in previous works, are well monodispersed and with an average diameter of ∼ 15 nm [44]. The statistical size parameters of the different AuNPs measured by TEM are reported in Table S2, together with the corresponding concentration of gold (*c*).

The chemical properties of the different types of AuNPs were investigated by SERS spectroscopy. The SERS spectra in Fig. 1D put in evidence the band associated to Au-CO groups around 2120 cm^−1^. Both the ligand-free AuNPs, synthesized by PLAL at the argon-water interface, and the citrate-stabilized nanoparticles lack gold-carbonyl groups on their surfaces. Although the SERS measurements don´t offer quantitative results, they clearly indicate the presence of carbon monoxide rich gold-nanoparticles (COR-AuNPs) when PLAL is performed at the air-water or CO_2_-water interface, and can distinguish them from the ligand-free AuNPs.

Using PLAL in water at different gas-water interfaces (air, argon and CO₂) enables us to control the surface chemistry of the produced AuNPs. When inert argon gas is in equilibrium with water, the only possible chemical reaction that occurs during PLAL is water splitting. This reaction is responsible for the partial oxidation of the AuNPs (by around 20%) [45, 46], resulting in ligand-free nanoparticles. When PLAL is performed at the CO₂-water or air-water interfaces, NaOH is used to enhance CO₂ solubility in water. Together with water splitting, the pulsed laser-driven CO₂ reduction reaction can also occur. This reaction is responsible for reducing aqueous CO₂ to CO, some of which is complexed with the gold surface [15, 47], creating carbon monoxide-rich gold nanoparticles (COR-AuNPs).

### Stability of the AuNPs in cell culture medium

**Fig. 2:**
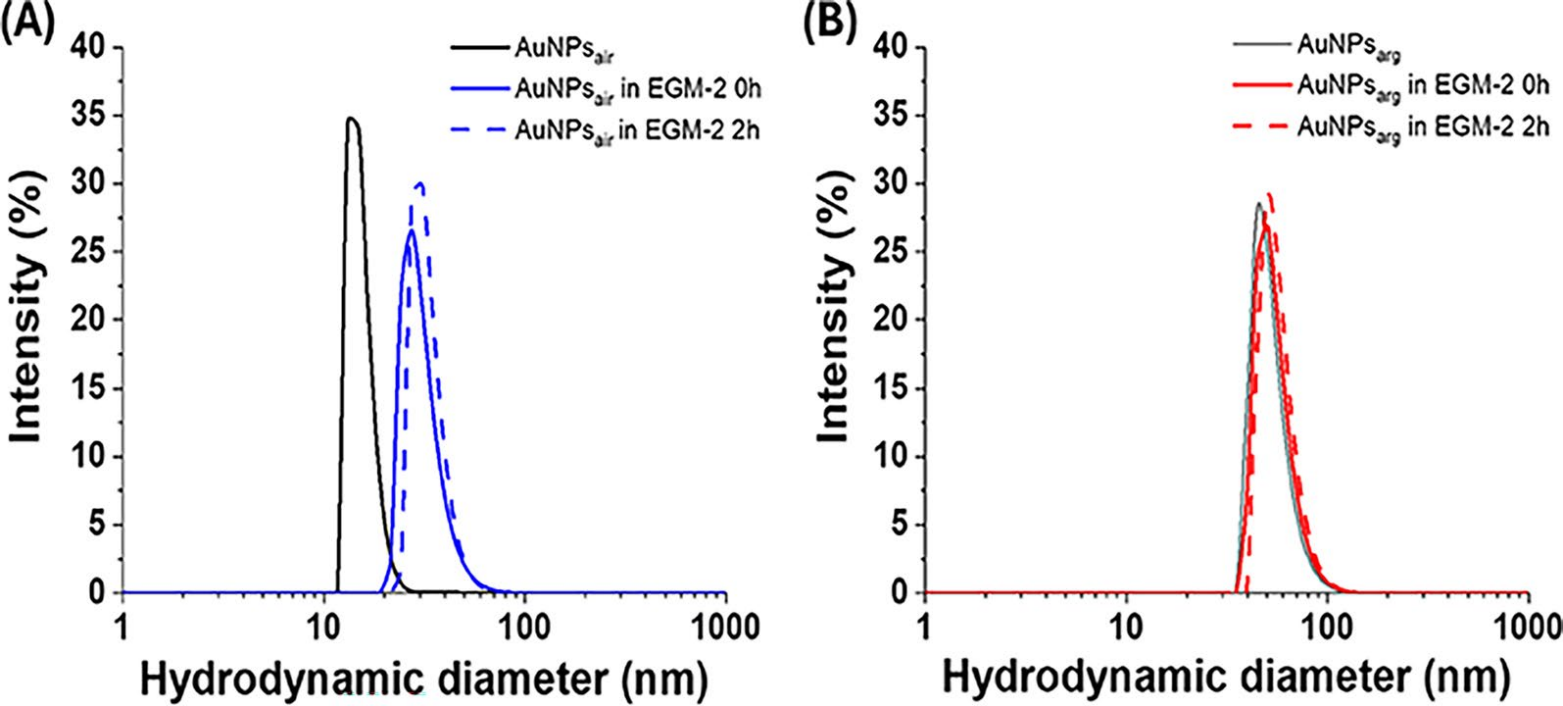
DLS investigation on the stability of the colloidal dispersion of AuNPs_air_ and AuNPs_arg_ in cell culture media. (**A**) Hydrodynamic diameter distributions of AuNPs_air_ in PBS solution (black solid line) and AuNPs_air_ dispersed in EGM-2 culture medium supplemented with 10% FBS measured immediately (blue solid line) and after 2 h (blue dotted line). (**B**) Hydrodynamic diameter distributions of AuNPs_arg_ (gray solid line) and AuNPs_arg_ in the presence of EGM-2 culture medium supplemented with 10% FBS measured immediately (red solid line) and after 2 h (red dotted line). In all tests, AuNPs_air_ and AuNPs_arg_ contained 1 mg/mL of PF127. The distributions represent the average of two measurements

In view of the biological application, the different AuNPs were further functionalized with the amphiphilic block copolymer Pluronic-F127 (PF127), to assure the stability of the colloidal systems in aqueous environment with high ionic-force (0.1 mol/L NaCl) [48] and favorite their cellular up-take [49]. In fact, in previous work [48] we observed that the addition of PF127 is responsible for the stability of the colloidal system after addition of salts in the water environment, where PF127 embeds the laser synthesized AuNPs forming gold-PF127 nanocomposites with dimensions ranging from 10 to a few hundreds of nm, which offers an additional steric stability to the system without changes on the shape of the LSPR spectra. The charge shielding effect of PF127 can be seen by observing the ζ-potential of different types of AuNPs before and after adding the copolymer to colloidal dispersions (see Table S2). Consistent with previous studies, the zeta potential of AuNPs synthesized by PLAL in water is negative, with the corresponding value being controlled by the pH of the water environment at the end of the synthesis process [46]. The sample characterized by the lowest pH of around 9.4 is AuNPs_CO_₂, which has the least negative potential of around − 34 mV compared with the other laser-synthesized nanoparticles. After the addition of PF127, the ζ-potential remains negative but decrease by between 10% and 90% depending on the gas-water interface used in the synthesis. Diluting the AuNPs with PF127 in DMEM with 10% FBS (pH 7.4) provokes a further decrease in the zeta potential, reaching values of approximately − 30 mV for AuNPs_air_ and AuNPs_arg_ and approximately − 4 mV for AuNPs_CO2_. In Fig. 2 we report the dynamic light scattering (DLS) measurements of AuNPs_air_ and AuNPs_arg_ in phosphate saline buffer (PBS) solution and in cell culture media along 2 h. For AuNPs_air_ in PBS we notice the presence of a band centered around 15 nm, which is shifted to ∼ 30 nm after the addition of the EGM-2 culture medium and FBS. Since the intensity of signal did not change upon time, the results indicated that the interaction of the gold-PF127 nanocomposites with the proteins of EGM-2-FBS culture media lightly changes the hydrodynamic size but preserves the stability of the colloidal system [50], with a final value of PDI of about 0.3.

In the case of AuNPs_arg_, the band in PBS is centered around 50 nm, showing agglomerates bigger than AuNPs_air_, which is consistent with the broadening of the extinction spectra reported in Fig. S1. In this case, we did not observe a significant shift of the band after the dispersion of the AuNPs in the cell culture media, and still the DLS signal was stable along two hours (at least), with a final value of PDI of about 0.2. Similar results were obtained also for the citrate stabilized gold nanoparticles and the AuNPs_CO2_.

### CO-release by the colloidal dispersions of COR-AuNPs

As recently reported in [15], the nanomaterial synthesized at the air-water or CO_2_-water interfaces contains CO rich nanoparticles exhibiting a SERS-active LSPR band, together with transparent photoluminescent organometallic nanocomposites (OMNCs). The OMNCs, which can be collected as supernatant after a proper centrifugation process, are composed of gold-nanoclusters and carboxylic acid salts, in particular sodium di-hidrated formate, sodium acetate and sodium lactate at the concentration of a few ppm, and originate from the pulsed laser driven chemical reduction of aqueous CO_2_. Analysis of the transparent OMNCs by SERS spectroscopy with films of silver nanoislands (SERSitive, Poland), did not show the typical Au-CO Raman resonance, confirming that the CO is not complexed on the photoluminescent organometallic nanocomposites. This observation is quite important to investigate independently the potential biological effect of the COR-AuNPs and the carboxylic acid based OMNCs. Moreover, total carbon measurements revealed that only exists a minor difference of less than 0.5 ppm in the carbon concentration of the pristine colloidal dispersion of AuNPs_air_ and the OMNCs. This observation suggests that the CO content on the COR-AuNPs synthesized at the air-water interface is less than 0.5 ppm, corresponding to about ∼ 20 µmolL^−1^.

For the experimental measurement of the CO content on the COR-AuNPs, it is necessary to investigate the CO-releasing properties of the nanoparticles. Some CORMs, such as the first generation CORM-2 and CORM-3, may release CO spontaneously in the plasma [51], while other CORMs only release CO intracellularly due to enzymatic trigger or ligand-exchange with proteins inside cells [52]. In the case of a gold surface, it is difficult to individuate the exact protein responsible for the triggering of CO release in an extracellular environment, since several biomolecules may have a great affinity to the metal, such as L-glutathione, cysteine, homocysteine and, most generally, the transmembrane proteins and all the thiol-ending neoglycoconjugates [53–55]. Since most of the works to induce extracellular CO release in CORMs by ligand-exchange make use of L-glutathione, we choose this protein for the test on the COR-AuNPs. Moreover, we also checked the potential release of CO in the DMEM with the addition of sodium dithionate, since some of the CORMs (i.e. CORM-3) are known to be unstable in the presence of physiological media containing cysteine [51, 56]. Unfortunately, the spectroscopic method based on carboxy-myoglobin detection on the Soret band [57–59] was not successful in both DMEM or water with L-glutathione and sodium dithionite as triggering species, as shown in Fig. S2. Details on the experimental procedures and comments on the spectroscopic results are reported in the Supporting Information.

**Fig. 3:**
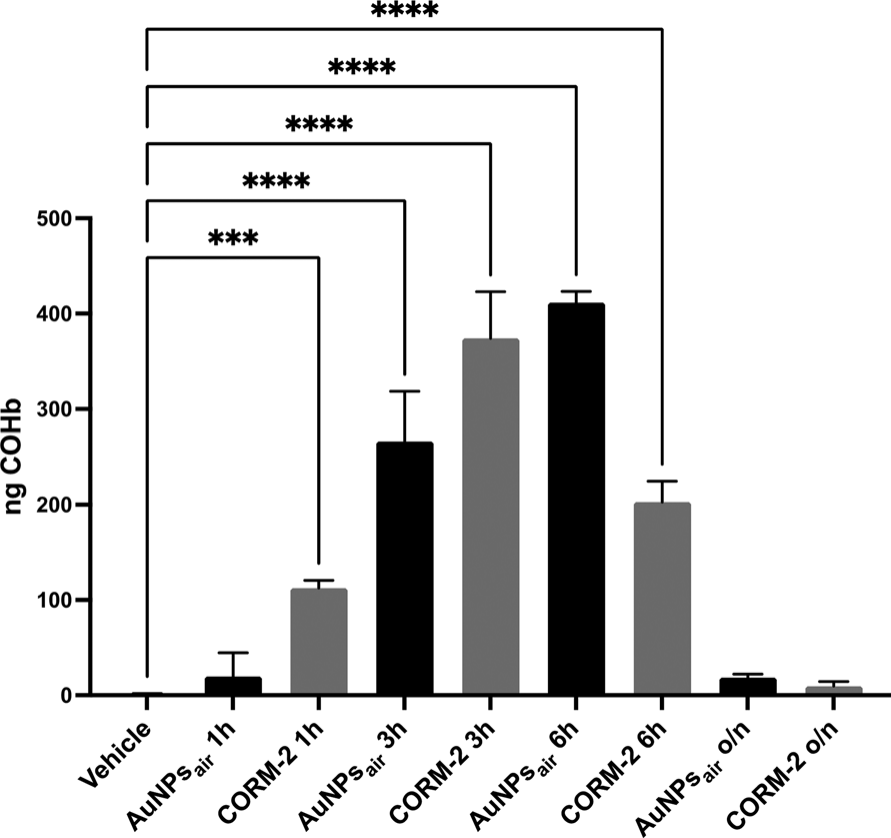
Quantification of CoHB released by K562 treated with CORM-2 or AuNPs_air_

To by-pass the difficulties encountered in the identification of the specific trigger for extracellular CO release of COR-AuNPs, the release of CO was directly measured intracellularly. To fulfill this objective, we employed human Chronic myeloid leukemia cells, K562, with the unique capacity to differentiate into pro-erythrocytes and produce hemoglobin. These cells were treated with 1µM Imatinib for five days to induce an erythroid phenotype characterized by globin expression and hemoglobin production [60]. Subsequently, the cells were exposed for different times to either 15 µg/ml AuNPs_air_ or 100µM of the well-known CORM-2 (Fig. 3). This concentration of CORM-2 was chosen in order that the mass of gold and ruthenium metal species was approximatively the same. Since the metal part of CORM-2 is composed by two ruthenium atoms for each molecule, a 100 µM solution of CORM-2 corresponds to about 20 ppm, which is very similar to the 15 ppm concentration of the COR-AuNPs.

Using this approach, we quantified carboxyhemoglobin (HbCO) formation in cell lysates (see Materials and Methods) to evaluate CO release. As expected, CORM-2 treatment resulted in a rapid and substantial increase in HbCO levels, with a clear peak at 3 h, reflecting its well-characterized CO-releasing kinetics. In contrast, AuNPs_air_ did not induce a significant HbCO formation after 1 h, indicating a delayed release profile. This result provides valuable insight into the differences in CO release between CORM-2 and COR-AuNP_air_. However, HbCO levels in AuNPs_air_ - treated cells gradually increased, reaching a peak at 6 h. Notably, at this time point, the levels of carboxyhemoglobin in cells exposed to AuNPs_air_ surpassed those observed with CORM-2. Following overnight (o/n) treatment with either CORM-2 or AuNPs_air_, HbCO levels declined to undetectable levels, suggesting that CO release from both sources is transient and becomes fully exhausted during the early phase of exposure.

These findings reveal distinct kinetics in the release and utilization of CO between CORM-2 and AuNPs_air_, underscoring a fundamental difference in their mechanisms of action. The rapid formation of carboxyhemoglobin (HbCO) in cells treated with CORM-2 within just 1 h confirms the immediate release of CO, consistent with the known properties of CORM compounds. This burst-like release likely results in a swift saturation of HbCO formation, as evidenced by the plateau observed shortly thereafter. In contrast, the delayed but sustained formation of HbCO in cells treated with AuNPs_air_ highlights a gradual and prolonged CO release mechanism and provides a more sustained and effective delivery of CO over time, potentially enabling prolonged biological effects. Notably, treatment for 6 h with either AuNPs_air_ or CORM-2 revealed that the effects on capillary morphogenesis were more pronounced and longer-lasting in ECFCs treated with AuNPs_air_ compared to those treated with CORM-2. This difference became even more significant following 24-hour treatment (Fig. S3).

### Pro-angiogenic properties of the CO-rich AuNPs

The objective of our investigation is to evaluate the pro-angiogenic properties of COR-AuNPs synthesized at the air-water interface in the presence of NaOH. Therefore, AuNPs_air_ were utilized in all biological and molecular assays. To further substantiate that the observed biological activity was specifically associated with the presence of carbon monoxide, the release of carbon monoxide has been measured in-vitro. Selected experiments were repeated employing CO-depleted AuNPs, such as chemically synthesized AuNPs (AuNPs_ch_) or AuNPs_arg_ as negative controls. To further validate this conclusion, we also utilized AuNPs_CO2_ synthesized at the CO_2_-water interface, known to be enriched in carbon monoxide.

**Fig. 4:**
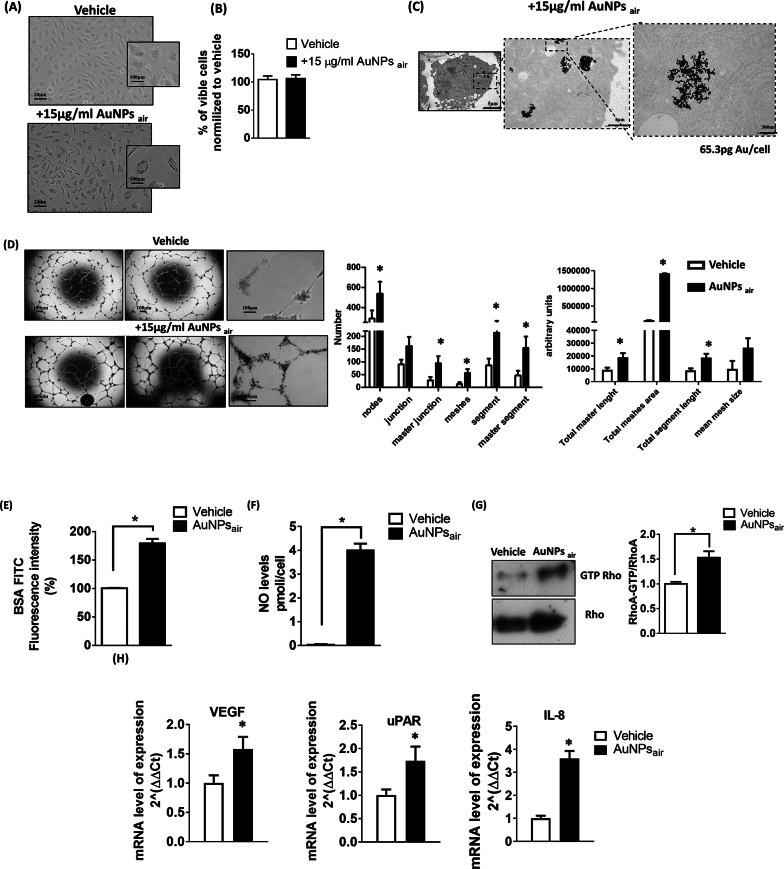
(**A)** Optical microscopy images of ECFCs treated with the vehicle or with AuNPs_air;_ (**B**) ECFC viability performed by trypan blue assay. (**C**) Transmission Electronic Microscopy Images of AuNPs_air;_ loaded ECFCS; (**D**) Capillary morphogenesis of ECFCs treated with the vehicle or AuNPs_sir;_. The capillary network was quantified by Angiogenesis Analyzer Image J tool. Histograms represent the mean number of number of nodes, junctions, master junctions, meshes, segments, master segment, total master and segment length, total mesh area and mesh size respectively. Representative microphotographs (x4 and x20) of capillary-like structures at 24 h are shown. Data are representative of measures obtained from at least nine fields. (**E**) Histograms show the permeability activity of ECFCs expressed as % of FITC-BSA cleared across the filter with respect to Vehicle-treated ECFC. (**F**) NO concentration in CM of vehicle- or AuNPs_air_-treated ECFCs. (**G**) Western blotting of total and GTP-loaded forms of small Rho-GTPase RhoA in control conditions and after ECFC treatment with AuNPs_air_. RhoA-GTP, GTP-loaded form of small Rho GTP-ase; RhoA, total un-loaded form of small Rho GTP-ase, used as a reference loading control. Histograms report band densitometry. Results are the mean of 3 different experiments performed in duplicate. (**H**) Real-time qPCR of angiogenic molecules such as VEGF, uPAR and IL8 in ECFCs treated with the vehicle or AuNPs_air_- Error bars: mean ± SD; *p < 0.05 indicates significant difference from each vehicle

To investigate the in vitro angiogenic potential of the different AuNPs, we employed ECFCs isolated from human umbilical cord blood (CB). Among the various endothelial cell models commonly used in angiogenesis research (e.g [61–63]).,, ECFCs represent a particularly relevant choice due to their high proliferative capacity, robust tube formation ability, and well-documented vasculogenic potential. These properties make ECFCs a reliable and physiologically relevant model for evaluating pro- or anti-angiogenic effects in vitro, especially in the context of therapeutic angiogenesis and regenerative medicine [64]. To this end a dose of 15ug/mL AuNPs_air_ was selected to treat ECFCs for 24 h, based on our preliminary data (Fig. S4). As reported in Fig. 4 A, B, C, ECFCs internalize AuNPs_air_ without eliciting cell toxicity. Further verification of NPs uptake by ECFCs and localization was performed by inductively coupled plasma atomic emission spectrometer (ICP-AES) and Transmission Electronic Micrscopy (TEM). Images at different magnification in Fig. 2 C show that AuNPs_air_ are internalized after 24 h incubation and are localized in vacuoles (i.e., endosomes and/or lysosomes) in the perinuclear region of the cells, just close to the Golgi complex. The intracellular concentrations of gold detected after 24 h incubation by ICP was quite impressive, 65.3 pg/cell as reported in Fig. 4C. Next, we assessed the ability of ECFCs-enriched AuNPs_air_ to perform capillary morphogenesis when cultured on Matrigel. Figure 4D, shows that ECFCs exhibit an enhanced capability to form elongated tubule-like structures after AuNPs_air_ treatment. Besides visible changes in appearance, the formation of the network has been quantified using several parameters [65], most notably the number and the length of nodes, master junctions and the number and the areas of formed meshes (right panel). Remarkably, AuNPs_air_ are retained inside the cells after having induced to differentiate. These results demonstrate that AuNPs_air_ treatment significantly boosts the total number of nodes, master junction and meshes thus improving vessel network formation.

It is well-documented that the presence of Mesenchymal Stem Cells (MSC) can significantly impact the behavior of endothelial cells and contribute to the development of functional vascular structures in the absence of exogenously added matrix proteins. Accordingly, to recent findings [66] we employed a 2D co-culture model to induce endothelial tubular network (ETN) formation by ECFCs in presence of the adipose MSC, wherein MSC and ECFCs were plated at a specific ratio of 5:1, respectively (Fig. S5). Endothelial network structures were formed when fibronectin fibrils were clearly visible by immunofluorescence 96 h following the direct co-culture of MSCs and ECFCs (Fig. S5 bottom panel). Interestingly, we observed a sharp increase of complete tubular structures when ECFCs were exposed to AuNPs_air_, as compared to ECFCs treated with control vehicle (Fig. S5). The 2D direct co-culture model confirms the enhanced propensity for ECFCs-enriched AuNPs_air_ to foster the formation of endothelial tubular networks.

Furthermore, we aimed to determine whether treatment with AuNPs_air_ could induce an increase in endothelial cell monolayer permeability utilizing fluorescein isothiocyanate (FITC**)** Bovine serum albumin (FITC-BSA) Transwell assay. To this end ECFCs treated with the vehicle or AuNPs_air_ were grown to confluence on Transwell filters. As illustrated in Fig. 4E, there was a discernible increase in FITC-BSA passage across the monolayer of ECFCs treated with AuNPs_air_ compared to unloaded ECFCs. Given the significant role of nitric oxide (NO) generation in vascular permeability regulation, we investigated whether NO might be involved in the endothelial permeability induced by AuNPs_air_. We found that AuNPs_air_ treatment boosted up the production of nitrite in ECFCs culture media (Fig. 4 F). Furthermore, AuNPs_air_-induced Rho activation (Fig. 4G) suggests a potential causal link to hyperpermeability response (Fig. 4E). These results are in agreement with other studies [67] showing that the activation of Rho-ROCK signaling increases the endothelial permeability caused by established proangiogenic factor VEGF-A. To expand our understanding of the broader implications of AuNPs_air_ treatment, we examined the expression of key angiogenic molecules within the treated cells. The augmented expression of angiogenic factors, including VEGF-A, uPAR, and IL-8, as demonstrated in Fig. 4H, underscores the potential angiogenic capacity of AuNPs_air_ at a molecular level. This aligns with the idea that AuNPs_air_ may not only influence monolayer permeability but also engage in intricate interactions with cellular signaling pathways associated with angiogenesis. The results presented collectively here offer a deeper insight into the effects of AuNPs_air_ on endothelial behavior. Indeed, the observed increase in permeability, coupled with the enhanced NO production and heightened expression of angiogenic molecules, collectively suggest that AuNPs_air_ might play a multifaceted role in promoting angiogenesis and vascular permeability.

### Convergence of angiogenic behavior between CO-rich AuNPs and VEGF

**Fig. 5:**
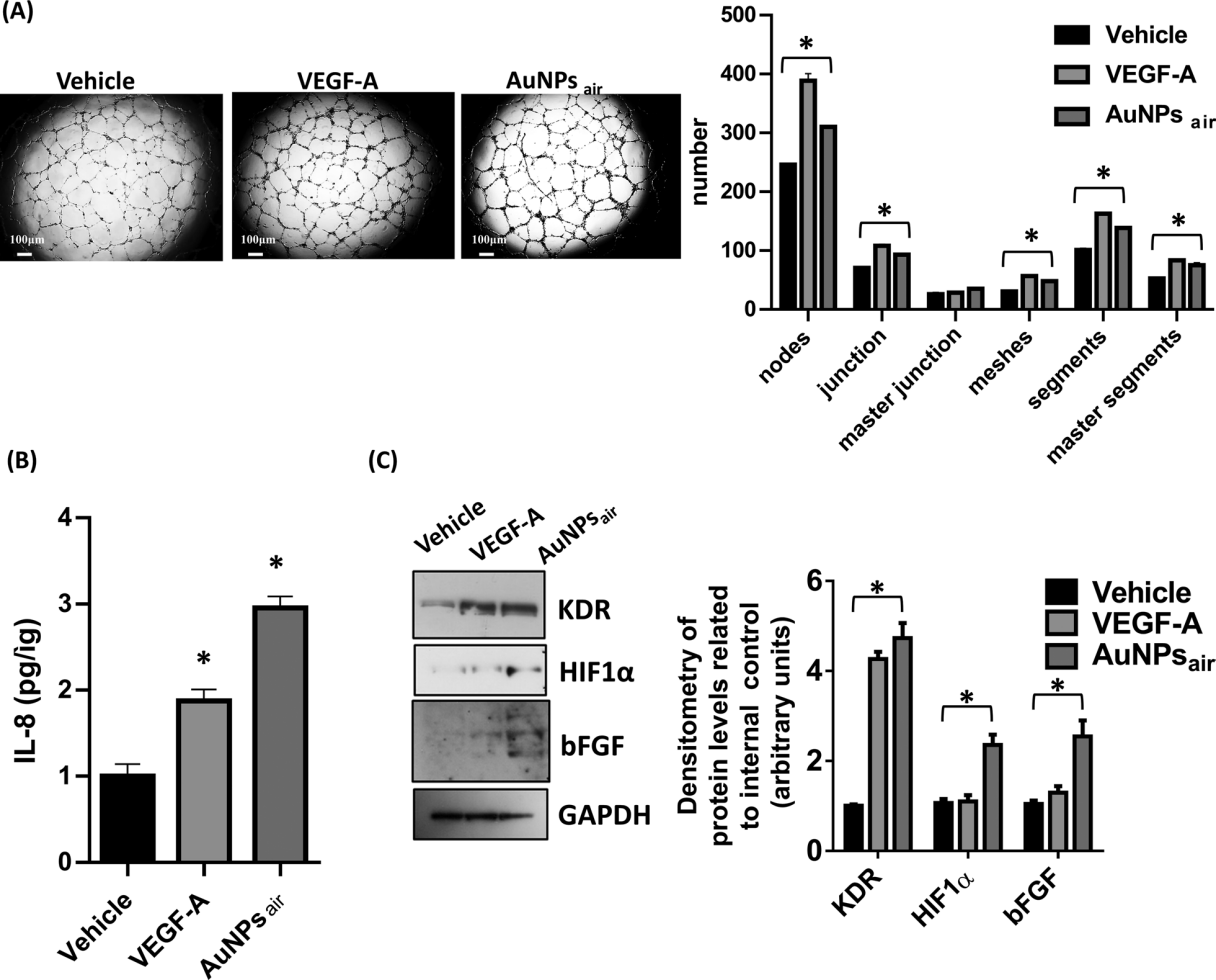
(**A**) Capillary morphogenesis of ECFCs treated with the vehicle, VEGF-A or AuNPs_air_. The capillary network was quantified by Angiogenesis Analyzer Image J tool. Histograms represent the mean number of number of nodes, junctions, master junctions, meshes, segments, master segments. Representative microphotographs (x4) of capillary-like structures at 24 h are shown. (**B**) ELISA of IL-8, released in CM of ECFCs treated with the vehicle, VEGF-A or AuNPs_air_ respectively. (**C**) Western blot analysis of important angiogenic cues (KDR, HIF1α, bFGF) in ECFCs treated with the vehicle, VEGF-A or AuNPs_air_. GAPDH is reported as loading control. Histograms report band densitometry. Results are the mean of 3 different experiments performed in duplicate. Error bars: mean ± SD; *p < 0.05 indicates significant difference from each vehicle

The potential translational significance of CO-rich AuNPs in enhancing angiogenesis was subsequently evaluated by conducting a detailed comparison of the biological and molecular effects of AuNPs_air_ with those elicited by vascular endothelial growth factor A (VEGF-A), a well-recognized primary driver of proangiogenic signaling pathways. Intriguingly, the assessment of capillary morphogenesis depicted in Fig. 5 A unveiled a striking parallel between the angiogenic response of ECFCs to AuNPs_air_ and VEGF-A stimulation. This equivalence was evident in the comparable number of generated tube-like structures and intricate networks. This surprising outcome underscores the potent proangiogenic potential of AuNPs_air_, highlighting its ability to evoke an angiogenic response that challenge that induced by the established proangiogenic factor, VEGF-A. At the molecular level, our investigations yielded unexpected insights. The quantity of interleukin-8 (IL-8) released into the culture media, as illustrated in Fig. 5B, was notably higher in cells treated with AuNPs_air_ compared to those treated with VEGF-A. Furthermore, our examinations revealed elevated protein levels of basic fibroblast growth factor (bFGF) and hypoxia-inducible factor 1 alpha (HIF1α) in cells treated with AuNPs_air_ when compared with VEGF-A treatment (Fig. 5 C). bFGF and HIF1α are recognized as pivotal players in orchestrating angiogenic processes. The increased expression of these molecules upon AuNPs_air_ treatment suggests that AuNPs_air_ might trigger a complex and diverse molecular response that extends beyond the scope of conventional angiogenic stimuli.

Remarkably, an increase in the total levels of vascular endothelial growth factor receptor 2 (VEGFR-2) was also noted following both AuNPs_air_ and VEGF-A treatments (Fig. 5 C). This observation raises the intriguing possibility that AuNPs_air_, akin to VEGF-A, might engage in mechanisms that enhance the cellular response to angiogenic cues through modulation of VEGFR-2 levels. This comparative study of AuNPs_air_ and VEGF-A effects unraveled a striking similarity in angiogenic response, with AuNPs_air_ eliciting equivalent capillary morphogenesis as VEGF-A. Furthermore, the divergent molecular effects observed upon AuNPs_air_ treatment highlight its capacity to influence angiogenesis-related factors in a distinct manner. These findings emphasize the multifaceted impact of AuNPs_air_ on proangiogenic signaling, suggesting promising avenues for its potential translational applications in therapeutic angiogenesis and regenerative medicine.

### Citrate stabilized AuNPs lack intrinsic angiogenic potential

**Fig. 6:**
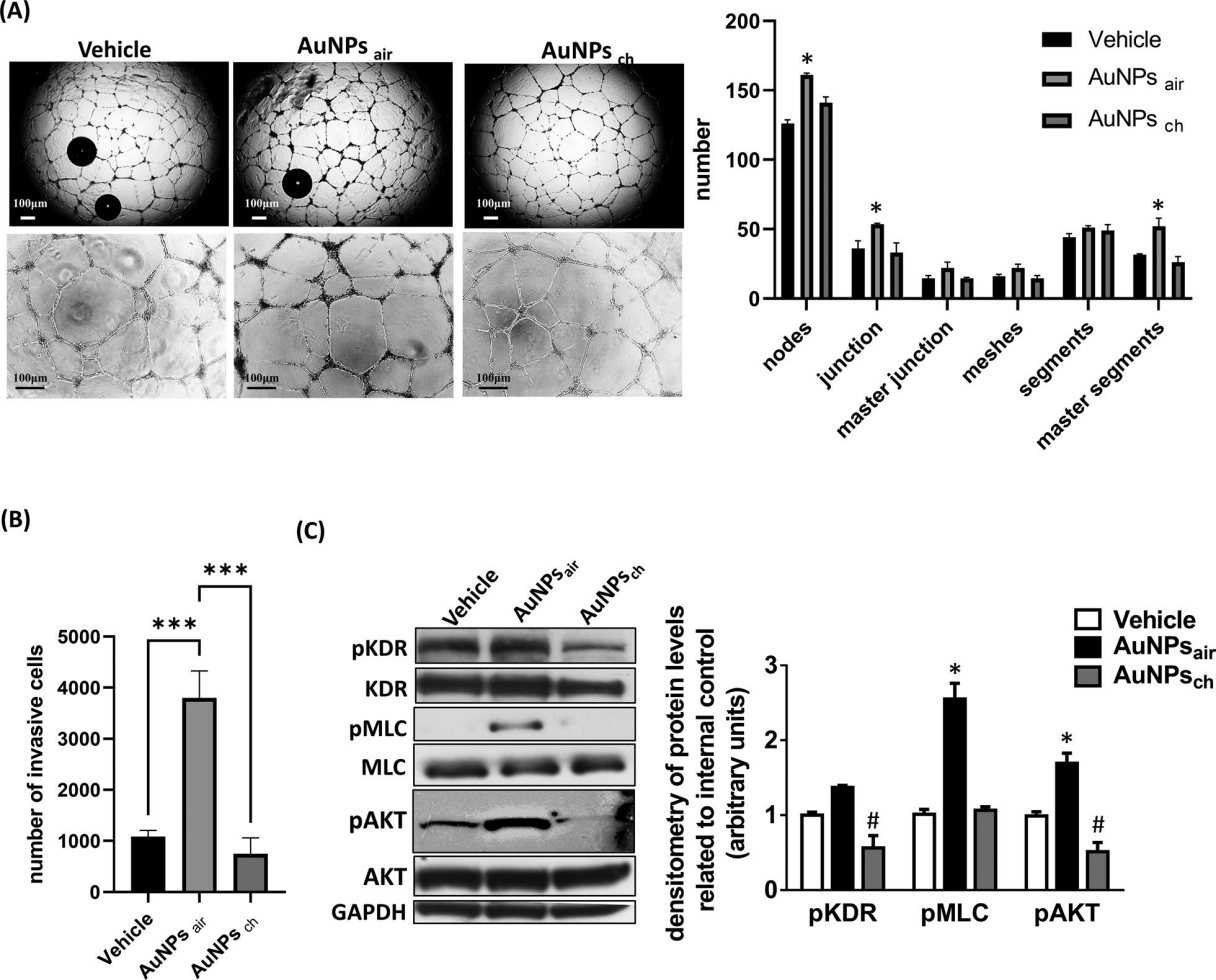
(**A**) Capillary morphogenesis of ECFCs treated with the vehicle, AuNPs_air_ or AuNPs_ch_. The capillary network was quantified by Angiogenesis Analyzer Image J tool. Histograms represent the mean number of number of nodes, junctions, master junctions, meshes, segments, master segment respectively. Representative microphotographs (x4 and x10) of capillary-like structures at 24 h are shown. Data are representative of measures obtained from at least nine felds. (**B**) Histograms refer to quantification of Matrigel invasion assay obtained by counting the total number of migrated cells/filter. (**C**) Western blot analysis in ECFCs treated with the vehicle, AuNPs_air_ or AuNPs_ch_. Histograms report band densitometry. Results are the mean of 3 different experiments performed in duplicate. Error bars: mean ± SD; *Asterisks*, significant difference (*, *P* < 0.05; ***p < 0.0001) of AuNPs_air_ samples from vehicle. *Number signs*, significant difference of AuNPs_ch_ (#, *P* < 0.05) from vehicle

To substantiate the hypothesis that the angiogenic behavior elicited by AuNPs_air_ was intricately linked to the Au-carbonyl groups on their surfaces, we conducted an in-depth exploration of the biological and molecular effects of the CO-depleted chemically synthesized citrate-capped gold nanoparticles, AuNPs_ch_. To this end, a series of experiments involving capillary morphogenesis and migration assays were undertaken using ECFCs treated with either AuNPs_ch_ or AuNPs_air_. The results, as illustrated in Fig. 6, revealed a compelling narrative. The quantitative analysis of vessel-like structures effectively validated the robust angiogenic potential of AuNPs_air_, and ruled out that this behavior could be ascribed solely to the elemental gold nature. Indeed, ECFCs treated with AuNPs_ch_ presented a less organized and mature web-like network, distinct from the robust and well-organized structures fostered by AuNPs_air_ treatment (Fig. 6 A). The trends identified in the capillary morphogenesis assay were reinforced by corresponding migration assays (Fig. 6B). The migration of ECFCs treated with AuNPs_air_ demonstrated a remarkable four-fold increase compared to cells treated with AuNPs_ch_. Importantly, both assays collectively corroborated the notion that AuNPs_ch_-treated ECFCs exhibited a diminished angiogenic profile when compared toAuNPs_air_ cells. This trend was also echoed at the molecular level, evidenced by the reduced levels of phosphorylated AKT and KDR (Fig. 6 C). This finding aligns with existing insights into the anti-angiogenic properties associated with gold nanoparticles synthesized through the chemical reduction method, as reported previously [68]. Conversely, the augmented phosphorylation levels of AKT, KDR, and the stimulated expression of phosphorylated myosin light chain (pMLC) in AuNPs_air_-treated ECFCs provided a striking contrast. Noteworthy, the observed phosphorylation of myosin light chain correlates consistently with the activation of the Rho pathway by AuNPs_air_ as reported in Fig. 6G. The observed increase of pMLC may account for the overexpression of the intracellular contractile control mechanisms required for an efficient vascular organization.

Overall, this molecular landscape underscored the dynamic engagement of the angiogenic signaling pathway under the influence of AuNPs_air_. By juxtaposing the divergent effects of AuNPs_ch_ and AuNPs_air_, our study represents a pivotal step in understanding the interplay between nanoparticle physical properties and angiogenic behavior.

### Unveiling the angiogenic mechanism orchestrated by the CO-rich AuNPs

**Fig. 7:**
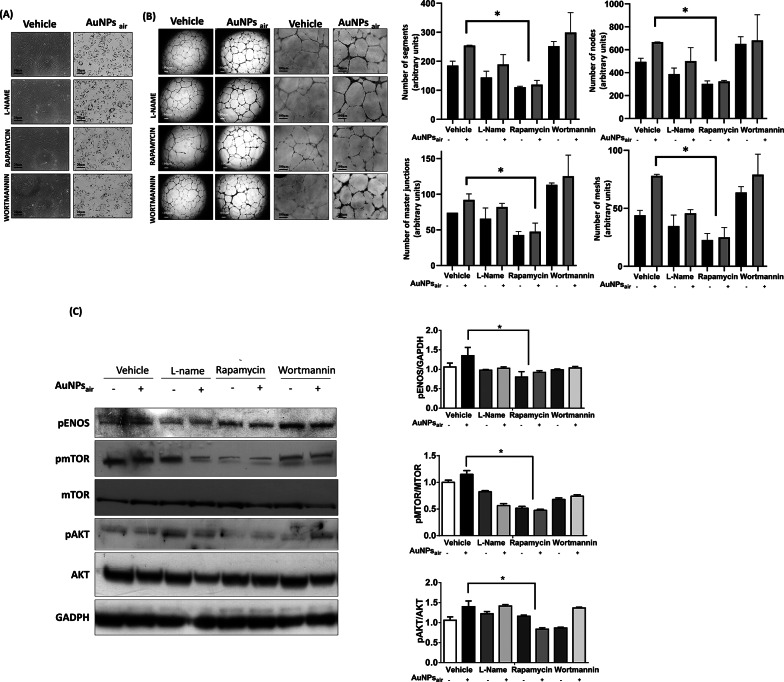
(**A**) Optical images of ECFCs treated with AuNPs_air_ in presence or absence of kinase inhibitors: L-name, Rapamycin and Wortmannin. (**B**) Capillary morphogenesis of ECFCs treated with the vehicle or AuNPs_air_ in presence of the kinase inhibitors. Histograms represent the mean number of segments, nodes, junctions and meshes respectively. Representative microphotographs (x4 and x10) of capillary-like structures at 24 h are shown. Data are representative of measures obtained from at least nine fields. (**C**) Western blot analysis of phosphorylated and total levels of eNOS, mTOR and AKT in ECFCs treated with AuNPs_air_ in presence or absence of kinase inhibitors. Histograms report band densitometry. GAPDH was also examined to ensure equal loading of samples in each lane. Results are the mean of 3 different experiments performed in duplicate. Error bars: mean ± SD; *Asterisks*, significant difference (*, *P* < 0.05) from vehicle

We next sought to determine the mechanism through which the CO-rich AuNPs orchestrate their potent angiogenic response. To achieve this, we subjected ECFCs to a spectrum of non-toxic concentrations of specific and non-specific inhibitors targeting various signaling pathways, as described in materials and methods and depicted in Fig. S6. Upon exposing these pre-treated cells to AuNPs_air_ (Fig. 7 A), we evaluated the impact on the angiogenic activation process, employing the capillary morphogenesis assay as a reliable indicator (Fig. 7B). Intriguingly, the results yielded a key insight. Among the spectrum of inhibitors tested, Rapamycin, a potent mTOR kinase inhibitor (Fig. 7 C), emerged as a pivotal regulator of the heightened angiogenic response induced by AuNPs_air_. Specifically, Rapamycin demonstrated the capacity to significantly restrain the enhanced angiogenic response induced by AuNPs_air_, a response that surpassed the effects of all other inhibitors (Fig. 7B). This revelation highlights the specificity of AuNPs_air_’s mechanism of action, further underlining its potential as a precise modulator of angiogenesis.

### Validation of carbon monoxide release role and chromatin modifying capability by CO-rich AuNPs

Driven by the recognition that rapamycin significantly reduces the angiogenic activity of astrocytes when exposed to the CO donor CORM-2, a reduction attributed to the decreased protein level of HIF-1 induced by CORM-2 [69], we were compelled to develop further experiments to associate the pro-angiogenic effect in ECFC to the intracellular CO release of the COR-AuNPs.

**Fig. 8:**
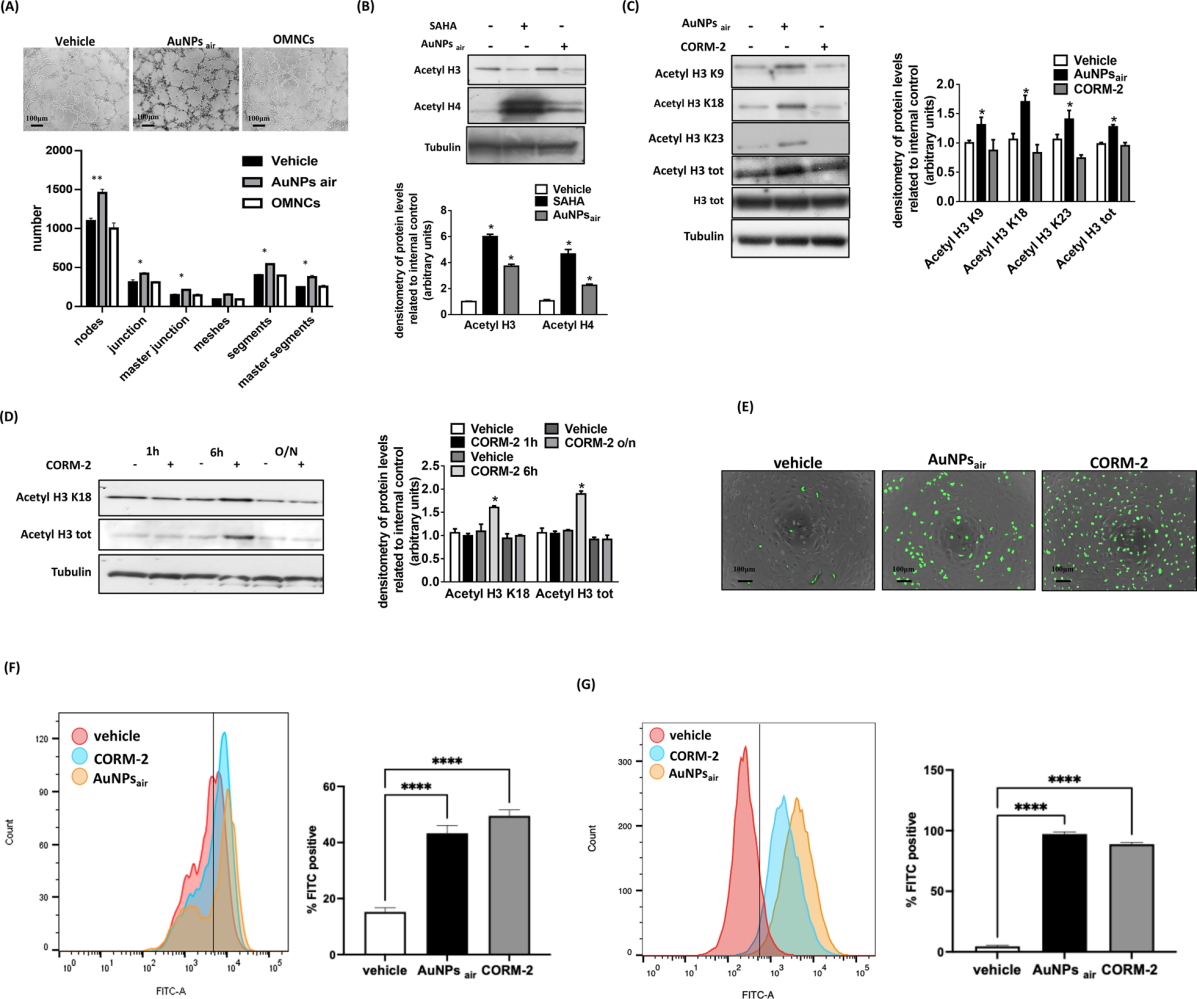
**A**) Capillary morphogenesis of ECFCs treated with the vehicle, AuNPs_air_ or OMNCs. The capillary network was quantified by Angiogenesis Analyzer Image J tool. Histograms represent the mean number of nodes, junctions, master junctions, meshes, segments, master segments. Representative microphotographs (x4) of capillary-like structures at 6 h are shown. (**B**-**D**) Western blot analysis of acetylation levels in ECFCs treated with SAHA (**B**), or AuNPs_air_ or CORM-2 (**C**-**D**) respectively. Histograms report band densitometry. (**E**-**F**) Nitric oxide detection was assessed with DAF-FM DA probe: fluorescent microscopy images showing NO production after treatment with AuNPs_air_ or CORM-2 for 6 h. Quantification of NO production was performed with flow cytometry. (**G**) Calcium flux was evaluated with Fluo 4 AM. Cells were treated with AuNPs_air_ or CORM-2 together with the probe for 1 h. Results are the mean of 3 different experiments performed in duplicate. Error bars: mean ± SD; *Asterisks*, significant difference (*, *P* < 0.0; **p < 0.001) from vehicle

In particular, to rule out a possible biological effect of the organometallic nanoclusters with acetic, formic and lactic acid, we performed capillary morphogenesis on ECFC cells treated with the OMNCs (Fig. 8 A) and did not observe any appreciable difference with the vehicle, confirming that the carboxylic acids present in the OMNCs don´t play a significant role in the enhanced angiogenesis, which is instead substantially linked to the intracellular CO release.

Given CO’s ability to modulate multiple genes by inducing global chromatin changes [70] and considering that selective histone deacetylase inhibitors (HDAC) like trichostatin A (TSA) induce endothelial cell migration, we subsequently evaluated whether AuNPs_air_ could induce chromatin remodeling in ECFCs. As illustrated in Fig. 8 C, the findings unveiled that AuNPs_air_ induced significant global acetylation of histone H4 and H3 in ECFCs, effectively positioning it alongside SAHA- an established HDAC inhibitor. We then proceeded to compare the acetylation levels of total histone H3 and specific lysine residues induced by AuNPs_air_ and CORM-2. Our findings revealed that AuNPs_air_ when administered overnight exhibited a greater capacity to induce heightened acetylation levels in ECFCs compared to CORM-2 (Fig. 8D). Examining the time course of H3 total and lysine 18 acetylation levels it becomes evident that CORM-2 initially induces an increase in acetylation within 6 h, followed by a subsequent return to baseline levels for H3 (Fig. 8E). This observation highlights that the effects induced by AuNPs_air_ on chromatin structure and function persist over an extended period, suggesting a sustained impact on epigenetic modifications. In agreement with previous studies, the effects of CORM-2 appear to be of a shorter duration in terms of their influence on chromatin acetylation. This finding underscores the dynamic nature of the cellular response to these two treatments, with AuNPs_air_ exerting a more prolonged, no toxic and potentially profound effect on the chromatin remodeling process. We want to emphasize that because the CO release and chromatin remodeling were observed in two different cellular models, the kinetics of these two biological processes only partially overlap. However, we observed a strong correlation between histone acetylation and the angiogenic response induced by AuNPs_air,_ which was further supported by measurements of NO production. Given the well-established role of NO as a key regulator of endothelial activation and vascular permeability- both critical processes in angiogenesis- as well as its function as an epigenetic modulator influencing chromatin architecture and gene expression, we assessed intracellular NO levels in ECFCs treated with AuNPs_air_ and compared them to those induced by CORM-2, a known NO donor. Fluorescence microscopy, fluorescence-based assays using a BioTek plate reader and flow cytometry (quantifying FITC-positive cells) consistently demonstrated that NO levels following overnight AuNPs_air_ treatment were comparable to those observed after 6 h of CORM-2 exposure (Fig. 8 E, F). These time points were selected based on evidence of maximal activity for both AuNPs_air_ and CORM-2, as indicated by chromatin remodeling (Fig. 8 C and D) and capillary morphogenesis (Fig. S3) in ECFCs- observed after overnight treatment with AuNPs_air_ and after 6 h with CORM-2 (Fig. 8D). Since it has been previously reported that in endothelial cells (ECs), eNOS activation can be induced via Ca²⁺-dependent dimerization and Akt-dependent phosphorylation, ultimately enhancing NO production and vascular function [71], we sought to investigate whether a similar mechanism is involved in our model. To this end, we performed calcium flux assays and observed that one hour of treatment with either CORM-2 or AuNPs_air_ led to a substantial increase in intracellular Ca²⁺ levels in ECFCs (Fig. 8G). These findings indicate that AuNPs_air_ activates the Ca²⁺/PI3K/Akt pathway, leading to eNOS-dependent NO production, which contributes to endothelial activation and angiogenesis. Moreover, the involvement of NO in modulating histone acetylation further supports a mechanistic link between AuNPs_air_- induced signaling and pro-angiogenic gene expression.

The confirmation of their role as a CO releaser, coupled with their enduring impact on chromatin remodeling, further underscores their many-sided mode of action in enhancing angiogenesis. It is of paramount importance to highlight that an overnight treatment utilizing 100µM CORM-2 yields a substantial 30% reduction in ECFC viability with respect to untreated and AuNPs_air_-treated cells (Fig. S7).

This result is not surprising, given that the cytotoxicity of CORM-2 is well understood and is known to be associated with the ruthenium core rather than the released carbon monoxide. For example, the heavy metal is responsible for antimicrobial activity, DNA damage in peripheral blood mononuclear cells, acute toxicity in human embryonic kidney cells and Madin-Darby canine kidney cells, and toxicity in primary rat cardiomyocytes [38].

### Surface chemistry-driven modulation of angiogenesis by PLAL-synthesized AuNPs in vitro and in vivo

Once demonstrated the clear differences in the biological activity of chemically synthesized citrate stabilized gold nanoparticles and AuNPs_air_, we performed further experiments to demonstrate the possibility to control the angiogenic effect of the PLAL synthesized AuNPs using different gas-water interfaces.

**Fig. 9:**
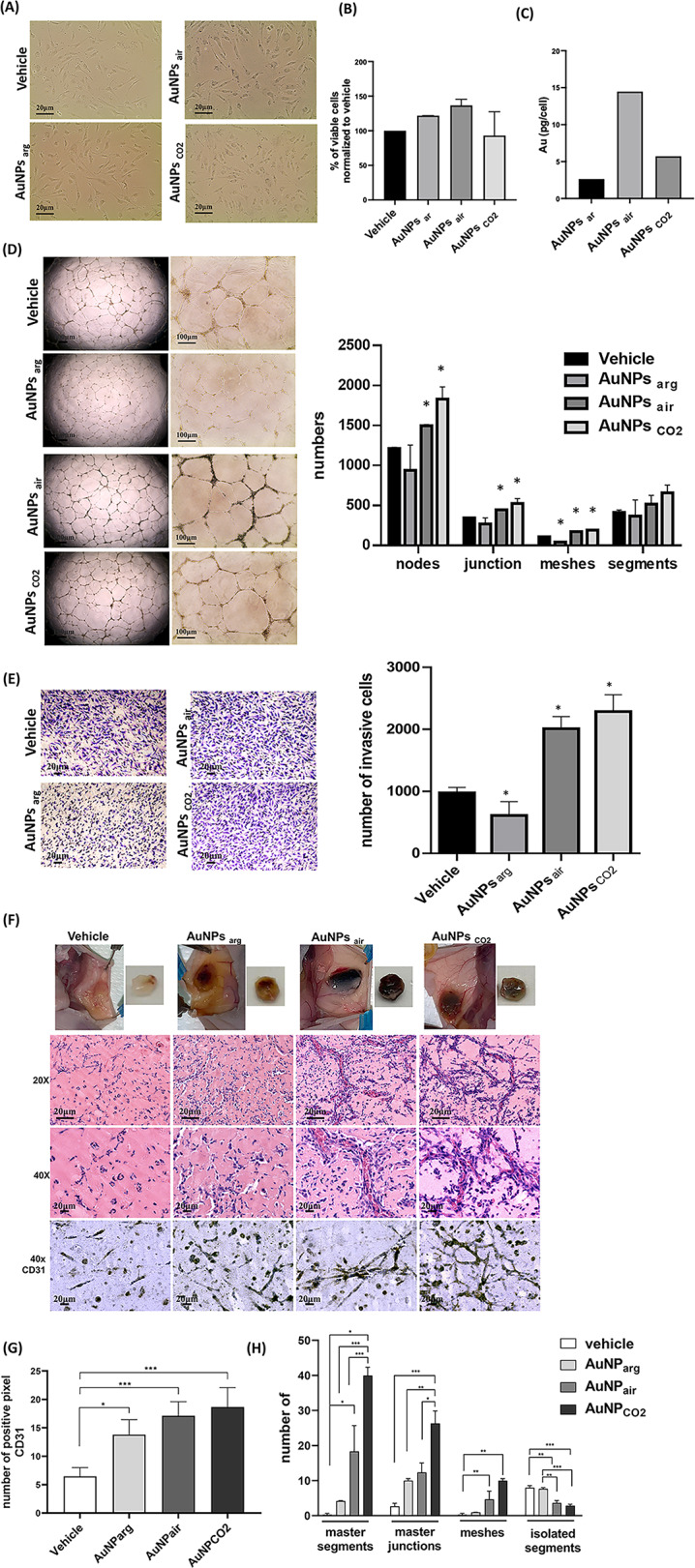
(**A**) Optical microscopy images of ECFCs treated with the vehicle or with AuNPs_arg_, AuNPs_air;_ or AuNPs_CO2_. (**B**) ECFC viability performed by trypan blue assay. (**C**)ICP-AES analysis of AuNPs loaded ECFCs. (**D**) Capillary morphogenesis of ECFCs treated with the vehicle or with AuNPs_arg_, AuNPs_air_ or AuNPs_CO2_. The capillary network was quantified by Angiogenesis Analyzer Image J tool. Histograms represent the mean number of nodes, junctions, meshes and segments. Representative microphotographs (x4 and x20) of capillary-like structures at 24 h are shown. Data are representative of measures obtained from at least nine fields. (**E**) Optical images of migrated cells. Histograms refer to quantification of Matrigel invasion assay obtained by counting the total number of migrated cells/filter. (**F**)Representative photographs of individual Matrigel sponges recovered at autopsy for the corresponding condition (Fig. 9 F upper panel) and histological analysis (Fig. 9 F lower panel) with Hematoxylin/eosin staining (x20 and x40). Error bars: mean ± SD; *p < 0.05 indicates significant difference from vehicle.Lower panel: representative images of immunohistochemical staining of CD31 expression on tumor sponges tissue sections. (**G**) The graph bar represents the densitometric analysis of the CD31 staining positive pixels. (**H**) Histograms illustrates the distribution of network patterns derived from angiogenesis parameters. The results are expressed as mean expression ± SEM. * *p* ≤ 0.05, *** *p* ≤ 0.001

For this purpose, we performed an array of in vitro angiogenic assays, including capillary morphogenesis and cell migration supplemented by an in vivo plug assay using ECFCs before and after treatments with distinct nanoparticles- namely AuNPs_air_, AuNPs_arg_, and AuNPs_CO2_ (Fig. 9). Ligand-free AuNPs_arg_, lacking gold-carbonyl groups on their surfaces, and AuNPs_CO2_, enriched with gold-carbonyl groups on their surfaces, respectively serve as negative and positive controls for assessing the biological activity of ECFCs. To confirm their differential CO-releasing capacity, we performed a carboxyhemoglobin (HbCO) assay in K562 cells treated for 6 h with AuNPs_air_, AuNPs_arg_, and AuNPs_CO2_, or CORM-2. The assay confirmed that only the CO-releasing nanoparticles- AuNPs_air_, AuNPs_CO2_ and CORM-2-led to measurable levels of carboxyhemoglobin, whereas AuNPs_arg_ did not induce any detectable HbCO formation (Fig. S8).

Intriguingly, Fig. 9 A visually revealed the substantial incorporation of AuNPs_CO2_ by ECFCs, followed by a lesser uptake of AuNPs_arg_. Importantly, there were no discernible toxic effects observed following the treatments (Fig. 9B), reinforcing the biocompatibility of these AuNPs. This microscopic observation was substantiated by the results of ICP-AES analysis presented in Fig. 9 C and Fig. S9, quantified as picograms of gold per cell. Turning to the crucial angiogenic test, capillary morphogenesis and migration assays demonstrated intriguing trends. The CO-rich AuNPs, specifically AuNPs_air_ and AuNPs_CO2_, exhibited a remarkably similar pro-angiogenic profile (Fig. 9D). In contrast, a noticeable inhibition of angiogenic activity manifested when ECFCs were subjected to the ligand-free AuNPs_arg_ (Fig. 9D and E). Moreover, consistent results were obtained through the Boyden chamber invasion assay (Fig. 9E). AuNPs_air_ and AuNPs_CO2_ induced a significant enhancement in Matrigel invasion by ECFCs, starkly contrasting with the minimal movement seen with AuNPs_arg_. These intriguing in vitro results were also corroborated by in vivo observations employing the Matrigel plug assay (Fig. 9F-H). Sponges soaked in ECFCs treated with AuNPs_air_ or AuNPs_CO2_ demonstrated a robust angiogenic response, characterized by an increased number of vascular structures with discernible lumens and red blood cells, contrasting the control plugs (ECFCs + vehicle) or plugs with ECFCs treated with. AuNPs_arg_. Histological analysis of angiogenesis, performed by immunohistochemical staining for CD31 in tissue sections, revealed a significant increase in CD31-positive cells and a marked enhancement of vessel density in mice injected with ECFCs + AuNPs_CO2_ or AuNPs_air_-treated ECs (Fig. 9 G and F). Remarkably, our data unequivocally highlight the potent pro-angiogenic properties of AuNPs_air_ and AuNPs_CO2_, and the inhibitory effects exerted by the ligand-free AuNPs_arg_ on the angiogenic process.

The results exclude the possibility that the intracellular gold content or nanoparticle size or charge may be correlated with the observed enhancement in the angiogenic activity. The data on ζ-potential reported in Table S2, highlight that the differences in the biological behavior observed for AuNPs synthesized at different gas-water interfaces, as well as for chemically synthesized AuNPs, are not associated to their surface charge.Interestingly, AuNPs_CO₂,_ which exhibit the strongest pro-angiogenic effects, were internalized at lower levels than AuNPs_air_ (Fig. 9c and Fig. S8), despite their enhanced activity. Notably, AuNPs_ch_, which exert anti-angiogenic effects, showed uptake levels comparable to the pro-angiogenic nanoparticles (Fig. S8),) suggesting that angiogenic modulation is independent of intracellular gold content or nanoparticle size. Observing Table S2, we also notice that the radius of AuNPs_CO₂_ (r∼3.3 nm) is almost three times higher than the radius of the ultrasmall AuNPs_air_ (r∼1.3 nm). Based on these results, in the case the radius of the AuNPs had an influence on their biological activity in ECFCs, we should expect a strong pro-angiogenic response of the AuNPs_arg_ or AuNPs_ch_, which have the highest dimension (r ∼7 nm). Vice versa, the ligand-free AuNPs_arg_ inhibits the angiogenesis (Fig. 9 D-I), coherently with results reported in literature on chemically synthesized CO-depleted gold nanoparticles (Fig. 6) [72, 73]. All these evidences definitively indicate that the observed angiogenic enhancement is independent of both intracellular gold concentration and nanoparticle size or charge, and is instead mostly associated to the presence of surface-bound gold-carbonyl groups, considering that the OMNCs show no effect on the angiogenesis process (Fig. 8 A). Finally, the distinctive intracellular CO-releasing properties of COR-AuNPs, compared to CORM-2, highlight their unique potential for biomedical applications. Unlike the rapid and transient CO release observed with CORM-2, COR-AuNPs exhibit a slower, more sustained CO release profile, which allows for prolonged modulation of cellular processes without the risk of acute saturation effects. This controlled release mechanism positions COR-AuNPs as a promising tool for therapeutic applications where gradual and sustained CO delivery is critical to achieving desired biological outcomes.

## Conclusions

**Fig. 10:**
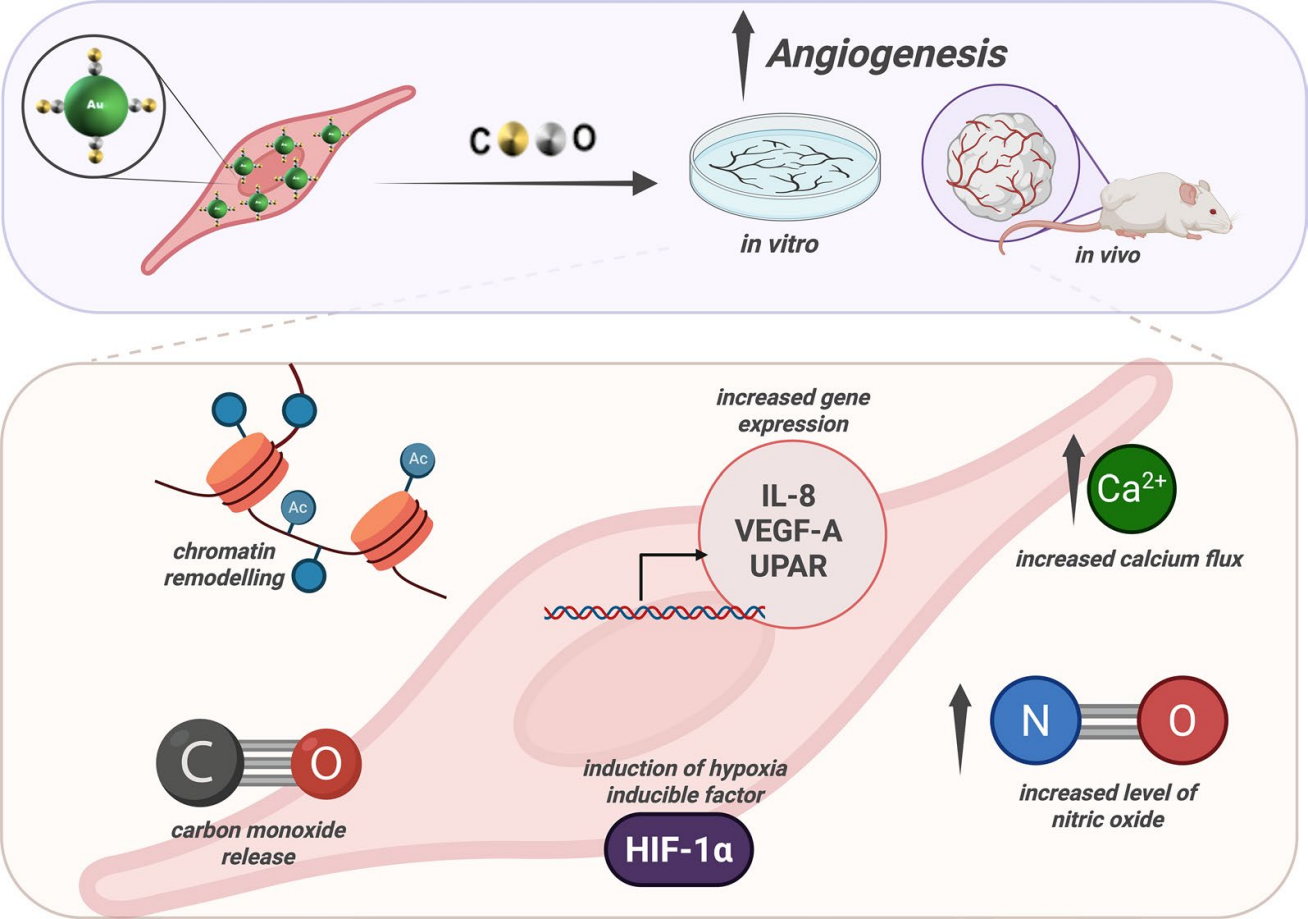
Schematic representation of the pro-angiogenic signaling pathways and molecular features induced by AuNPs_air_ in ECFCs. The illustration, created with BioRender, summarizes the key intracellular mechanisms triggered by AuNPs_air_ treatment, including Ca²⁺ influx, activation of the PI3K/mTOR/Akt pathway, NO production, and subsequent effects on histone acetylation and endothelial activation leading to angiogenesis

In this work we have shown that it is possible to control the biological activity of AuNPs loaded in a particular class of EPCs known as ECFCs, by the proper choice of the gas-water interface during pulsed laser ablation of a gold target. While ligand-free AuNPs produced at the argon-water interface show an anti-angiogenic behavior, CO-rich AuNPs (< 20 µmol/L of CO) produced by the pulsed laser driven CO_2_ reduction reaction are characterized by an intracellular release of CO by gold-carbonyl groups formed in the ablation process, ultimately triggering a cascade of events that stimulate neo-angiogenesis by ECFCs in a frame of total biocompatibility. CO-rich AuNPs induce a process of capillary morphogenesis in vitro, the formation of evident vascular structures and the formation of vessels in the Matrigel-plug model in nude mice (Fig. 10). The mechanism involves the CO-dependent activation of Rho, a small GTPase protein. This enhancement initiates the phosphorylation of eNOS through the PI3K/pAKT/mTOR pathway, resulting in increased production of NO, heightened vascular permeability, activation of HIF-1alpha, and ultimately, angiogenesis. Furthermore, CO-induced chromatin modification, particularly increased acetylation of histone H3, is observed in ECFCs treated with AuNPs. Our findings align with previous studies linking CO-dependent chromatin modification to the regulation of gene expression responsible for endothelial cell migration and angiogenesis (Fig. 10). In comparison to traditional CORMS, the laser synthesized COR-AuNPs can be stored for months on the laboratory shelf, eliminating the difficulties associated to oxidation by the environment. Notably, our findings demonstrate that AuNPs_air_ support a non-inflammatory and non-thrombogenic endothelial phenotype (Fig. S10), essential for the biosafety and translational viability of nanomaterials intended for vascular applications. ELISA and Western blot analyses revealed that ECFCs exposed to AuNPs_air_maintained low levels of pro-inflammatory cytokines and adhesion molecules (IL-6, IL-1α, IL-1β, GM-CSF, MCAF/MCP-1, ICAM-1, VCAM-1), while upregulating Thrombomodulin, a key anti-coagulant and anti-inflammatory marker. In contrast, AuNPs_ch_ and CORM-2 treatments induced higher expression of inflammatory cytokines, suggesting a less favorable endothelial response.

Nontheless, these results underscore the importance of preserving endothelial quiescence to ensure compatibility and therapeutic efficacy, further highlighting the translational potential of AuNPs _air_ in vascular nanomedicine.

Moreover, it is our intention to investigate in the future the possibility of the chemical functionalization of the COR-AuNPs to obtain a targeting in specific sites of interest. Looking ahead, the use of nanoparticles (NPs) with angiogenic potential, including those discussed in this study, presents promising avenues for overcoming challenges posed by the complex organ structure and native microenvironments. In conclusion, the potential for vascular delivery, supported by advances in ultrasound-guided cannulation techniques, presents a valuable opportunity for targeted therapeutic interventions across multiple organ systems. Recent study underscores the importance of developing vascular-compatible nanomaterials and biomimetic delivery systems [25] capable of achieving organ-specific biodistribution [74] with minimal systemic toxicity. In this context, membrane-coated technologies, including cell membrane-camouflaged nanoparticles, represent a cutting-edge approach that mimics natural biological interfaces to enhance circulatory stability, immune evasion, and targeting specificity [25, 74]. Moreover, the integration of natural polymers, such as hydroxyethyl starch and its derivatives-known for their excellent biocompatibility and biodegradability-further improves the safety profile and translational potential of these nanocarriers [26]. Collectively, these strategies define a promising future for vascular nanomedicine, enabling safer, more efficient, and clinically viable therapeutic delivery through biomimetic and physiologically adaptive platforms.

These advancements underscore the potential of our findings in contributing to the development of innovative strategies for angiogenesis modulation in therapeutic applications.

## Experimental section

### Synthesis of the AuNPs

AuNPs were synthesized by both wet-chemical methods and Pulsed Laser Ablation in water (PLAL). In the first case, citrate stabilized AuNPs (AuNPs_ch_) were obtained by the Turkevich method [35], similarly as reported in our previous works [36, 40]. PLAL was performed in deionized water in equilibrium with different gaseous environment at the pressure of 1.0 Atm: argon (AuNPs_arg_), air (AuNPs_air_), or a mixture of argon (99%) with CO_2_ (1%) (AuNPs_CO2_, percentages relative to the partial pressure). In the last two cases, AuNPs_air_ and AuNPs_CO2_, NaOH was introduced in the water environment before the ablation to enhance the CO_2_ solubility, at the concentration of 4 mmol/L and 15 mmol/L, respectively.

PLAL was performed using a laser source Q-Smart 850 (Quantel, U.S.A.) emitting laser pulses with a temporal duration of 5.8 ns, repetition rate of 10 Hz, and wavelength of 532–1064 nm. A gold target from Kurt&Lesker company (purity better than 99%) was immersed in 8 ml of the liquid, and irradiated for a total time of 6 h (2.16 × 10^5^ laser pulses) after focusing the laser pulses by the use of a lens with focal length of about 15 cm.

At the end of the PLAL process, the obtained colloidal dispersion of NPs was mixed with the amphiphilic block copolymer Pluronic-F127 (PF127) at the concentration of 1 mg/ml, similarly as reported in [41]. Before the interaction with the cells, the AuNPs were filtered by pore diameters of about 0.22 μm (Syringe Filters-K18-230, KASVI), to minimize the presence of environment contaminants and/or microorganisms.

The AuNPs_arg_ and AuNPs_CO2_ were synthesized using pulses at the wavelength of 532 nm, while in the case of AuNPs_air_, PLAL was instead performed by simultaneous irradiation with laser pulses at the wavelength of 532 nm and 1064 nm. Complete details of the experimental set-up used in this particular configuration are reported in [15]. The values of the dimension, energy and fluence of the laser pulses in the different configurations, are reported in Table S1.

### Characterization of the AuNPs

The synthesized AuNPs were characterized by dynamic light scattering (DLS), UV-Vis spectroscopy, surface enhanced Raman spectroscopy (SERS), inductively coupled plasma mass spectrometry (ICP-MS), total carbon and transmission electron microscopy (TEM).

The effect of the copolymer PF127 on the stability of the colloidal dispersion of nanoparticles in culture media was studied by DLS for the AuNPs synthesized at different gas-liquid interfaces. The particle size was characterized using the numerical distribution as a function of the hydrodynamic diameter. The analysis was carried out using an SZ-100 analyzer from Horiba Instruments (Kyoto, Japan). The analyses were conducted at 25 °C, with a scattering angle of 90°, in duplicate and lasting 120 s, using a 10 mW laser with a wavelength of 532 nm. For the analysis, the AuNPs were dispersed in PBS or in EGM-2 + 10% FBS culture medium at ratios of 3:1 and 5:1 (nanoparticle: culture medium) for AuNPs_air_ and AuNPs_arg_, respectively. AuNPs_air_ and AuNPs_arg_ containing 1 mg/mL of the amphiphilic block copolymer Pluronic-F127 (PF127) were used for all tests. Before analysis, the samples were filtered through syringe filters with a pore diameter of 0.22 μm (Syringe Filters-K18-230, KASVI) to remove possible contaminants and then subjected to ultrasound for approximately 3 min. Measurements were taken immediately after preparation and again after 2 h of incubation. The autocorrelation functions obtained from the colloidal dispersions were fitted using HORIBA NextGen Project SZ-100 software for Windows, resulting in the hydrodynamic diameter distributions.

The extinction spectra of the AuNPs were measured by a double beam spectrophotometer model Lambda 950 (Perkin Elmer, U.S.A). SERS was performed using a Raman microscope model XploRA (HORIBA), with an excitation wavelength of 638 nm, and used to verify the presence of gold-carbonyl groups on the surface of the nanoparticles. The samples were prepared by drying 6 drops of the fresh nanomaterial (40 µL per drop) at a temperature of 40 °C over a clean glass substrate, similarly as reported in [75]. For the quantification of the gold concentration by ICP-MS we used a spectrometer model Nexlon 300X (Perkin Elmer, U.S.A). Total carbon (TC) was measured using a Shimadzu analyzer (model TOC-VCPN, Japan) with a resolution of 0.1 ppm.

The average and standard deviation of the statistical size distribution of the AuNPs were measured using a JEOL 2100 F electron microscope in transmission mode, operated at an electron acceleration voltage of 200 kV.

DLS was performed using the ZetaSizer instrument model Nano-ZS from Malvern (United Kingdom), operating at the wavelength of 633 nm. Before the measurements, the AuNPs were filtered using filters with a pore diameter of 0.22 μm (Syringe Filters-K18-230, KASVI) to remove any contaminant that could significantly interfere with the measurement. The analysis was also carried out on AuNPs in DMEN culture medium (Dulbecco’s Modified Eagle’s Medium - high glucose, Sigma-Aldrich^®^) supplemented with 10% fetal bovine serum (FBS, Gibco™), in which case a 0.5mL aliquot of the AuNPs colloidal dispersion was added to the cuvette containing 0.5mL of culture medium.

### Preparation of ECFCs and cell culture

For the angiogenic studies we selected ECFCs among the various Endothelial Cell models available, owing to their high proliferative capacity, stable endothelial phenotype, and strong tube-forming and vasculogenic potential [61–63]. These features make ECFCs particularly suitable for investigating endothelial function and vascular regeneration. ECFCs, a subpopulation of Endothelial Progenitor Cells (EPCs), were isolated from > 50 mL human UCB of healthy newborns, as described in [4, 76] after maternal informed consent and in compliance with Italian legislation, and analyzed for the expression of surface antigens (CD45, CD34, CD31, CD105, ULEX, vWF, KDR, and uPAR) by flow-cytometry. The purification and use of stem cells from cord blood for research purposes is permitted by an Italian law after obtaining informed consent R711-D from the mothers (art. 2, paragraph 1, letter f, decree of 18 November 2009). ECFCs were cultured in EGM2 medium (Euroclone) supplemented with 10% (v/v) fetal bovine serum (FBS, Euroclone). Cells were incubated at 37 °C with 5% CO_2_ saturation.

### Cellular viability and uptake

ECFCs, were seeded in 6 cm dishes at a density of 2 × 10^5^ cells in a humidified atmosphere with 5% CO_2_ and then incubated in the appropriate culture media (EGM2)) (3 mL per well) containing AuNPs at 15 µg/mL for 24 h. Cells cytotoxicity was determined with trypan blue staining: 20 µL of cell suspensions were resuspended with an equal volume of 0.4% (w/v) trypan blue solution prepared in 0.81% NaCl and 0.06% (w/v) dibasic potassium phosphate. Viable and non-viable cells (trypan blue positive) were counted separately using a dual-chamber hemacytometer and a light microscope.

Optical microscopy was used to assess qualitative intracellular uptake of AuNPs. Cellular images were acquired through the EVOS xl core microscope (AMG, Advanced Microscopy Group).

**Transmission electron m icroscopy** (**TEM) analysis of AuNPs-enriched ECFCs**

To investigate the uptake and intracellular localization of nanoparticles, we performed transmission electron microscopy (TEM) analysis. ECFCs were seeded in 6-well plates at a density of 1.5 × 10^5^ cells per well and allowed to reach 70% confluence. Next, cells were incubated with culture medium (2 mL per well) containing suspensions of AuNPs at a concentration of 15 µg/mL for 24 h, then collected by trypsin treatment, and centrifuged at 1000 rpm for 5 min in a 1.5 mL Eppendorf tube. The cellular pellet was then fixed in isotonic 4% glutaraldehyde and 1% OsO4, dehydrated, and embedded in Epon epoxy resin (Fluka, Buchs, Switzerland) for electron microscopy. Ultrathin sections were stained with aqueous uranyl acetate and alkaline bismuth subnitrate, and viewed and photographed under a JEM 1010 transmission electron microscope (Jeol, Tokyo, Japan) equipped with a MegaView III high-resolution digital camera and imaging software (Jeol).

**Inductively coupled plasma atomic emission spectroscopy (ICP-AES)**

To quantify gold uptake, we performed analysis ICP-AES. ECFCs were seeded in 6-well plates at a density of 1.5 × 10^5^ cells and allowed to attach overnight. On the next day, cells were incubated with culture medium containing AuNPs at a concentration of 15 µg/mL for 24 h. Cells were then washed two times with PBS (Invitrogen), detached with a trypsin treatment, and counted using a hemocytometer. The Au concentration in cells was measured by a Varian 720-ES Inductively Coupled Plasma Atomic Emission Spectrometer (ICP-AES) equipped with a CETAC U5000 AT + ultrasonic nebulizer, this latter allowing to increase the method sensitivity. Cellular pellets were digested in a thermo-reactor at 80 °C for 6 h with 500 µL of concentrated Suprapur HNO3 obtained by sub-boiling distillation. Every sample was then diluted to a final volume of 5.0 mL and spiked with 1 ppm of Ge used as an internal standard before the analysis. Calibration standards were prepared by gravimetric serial dilution from a commercial standard solution of Au at 1000 mg/L. The operating conditions were optimized to obtain maximum signal intensity, and between each sample, a rinse solution of 1% v/v HNO3 Suprapur grade was used in order to avoid any “memory effect”.

### Cell treatments with pAKT, pMTOR specific inhibitors

To identify the cell signaling pathways involved in AuNPs_air_-mediated effects, we employed a pharmacological approach using specific inhibitors targeting key pathways such as mTOR, AKT, followed by Western blot analysis to assess pathway modulation.

Inhibition of PI3K/pAKT/mTOR pathway in vitro was performed with specific inhibitors. Rapamycin (HY-10219), a potent and specific mTOR inhibitor, was used at 100nM, Wortmannin (HY-10197), a selective and irreversible PI3K inhibitor was used 25nM and L-name Hydrochloride (HY-18729 A), that inhibits nitric oxide synthase (NOS) activity was used 1mM. All inhibitors were purchased from MedChemExpress (LCC, Monmouth Junction, NJ, USA). ECFCs were seeded in 60 mm petri dish at a density of 3 × 10^5^ cells and treated o/n with inhibitors and AuNPs at final concentration of 15 µg/mL before performing in vitro capillary morphogenesis and WB analysis.

### Western blot analysis

Harvested cells were resuspended in RIPA buffer (pH 7.4) (Merk Millipore, Vimodrone, MI, Italy) containing a cocktail of proteinase inhibitors (Calbiochem, Merck, Darmstadt, Germany) and treated by sonication (Microson XL-2000, Minisonix, Farmingdale, NY, USA). Aliquots of supernatants containing equal amounts of protein (40 µg) in Laemmli buffer were separated on Bolt Bis-Tris Plus 4–12% precast polyacrylamide gels (Life Technologies, Monza, Italy). Fractionated proteins were transferred from the gel to a PVDF nitrocellulose membrane using an iBlot 2 system (Life Technologies, Monza, Italy). Blots were stained with Ponceau red to ensure equal loading and complete transfer of proteins, and then blocked for 1 h at room temperature with with 6% skimmed milk in PBS containing 0.1% Tween. Subsequently, membranes were probed at 4 °C overnight with specific primary antibodies: RhoA (Mouse monoclonal 1:800 Millipore, Cat#05-778, Lot#3064837); HIF-1α (D5F3M) (Mouse monoclonal 1:500 Cell Signaling Cat#79233, Lot#1); FGF-2 (bFGF) (Mouse monoclonal 1:500 Santa Cruz Biotecnology, Cat# 74413, Lot#K2311); pKDR/pVEGFR2 (Rabbit polyclonal 1:1000 Cell Signaling, Cat#2478, Lot#13); KDR/VEGFR2 (Rabbit polyclonal 1:1000 Cell Signaling, Cat#9698, Lot#4), pMLC2 (Rabbit polyclonal 1:1000 Cell Signaling, Cat#3671, Lot#7); MLC2 (Rabbit polyclonal 1:1000 Cell Signaling, Cat#3672, Lot#6); pAKT and AKT (rabbit, polyclonal 1:1000, Cell signaling Technology, pAKT Cat#9271, Lot#14; AKT Cat#9272, Lot#28), pmTOR (Ser2448) (rabbit polyclonal 1:1000 Cell signaling Technology, Cat#5536, Lot#12), mTOR (rabbit polyclonal 1:1000 Cell signaling Technology, Cat# 2972, Lot#10), VCAM-1 (rabbit, polyclonal 1:1000, Cell signaling Technology, Cat#13662, Lot#4), CD-45/ICAM-1 (rabbit, polyclonal 1:1000, Cell signaling Technology, Cat4915, Lot#4), CD141/Thrombomodulin (rabbit, polyclonal 1:1000, Cell signaling Technology, Cat#43514, Lot#1.); α-tubulin (rabbit polyclonal 1:1000 Cell Signaling, Cat# 2144, Lot#8) and GAPDH (rabbit polyclonal 1:1000, Cell signaling Technology) and 𝛼-tubulin (rabbit polyclonal 1:1000 Cell Signaling, Cat# 2144, Lot#8) used to assess equal amounts of protein loaded in each lane. Antirabbit IgG (whole molecule)–Peroxidase antibody (1:5000 Cell Signaling, Cat#7074P2, Lot#33) or antimouse IgG (whole molecule)–Peroxidase antibody (1:5000 Invitrogen, Cat#A16084, Lot#66-119-121520) were used as secondary antibodies; the enhanced chemiluminescence (ECL) procedure was employed for development.

### RhoA activity assay

RhoA is a small signaling G protein (small GTPase) belonging to the Ras superfamily, involved in regulating the cytoskeleton, cell shape, motility, and various cellular processes. Measuring RhoA activity provides important insights into the molecular mechanisms underlying angiogenesis, particularly in response to AuNPs air treatment.

For the assay cells from different experimental conditions (control, AuNPs) were lysed in radio-immunoprecipitation assay buffer, the lysates were clarified by centrifugation, and RhoA GTP was quantified. Briefly, lysates were incubated with 10 µg rhotekin–glutathione S-transferase (GST) fusion protein (Millipore) or p21-activated kinase-GST fusion protein, both absorbed on glutathione–Sepharose beads for 1 h at 4 °C. Ratios between activated (GTP-bound) RhoA adsorbed to the beads were quantified by Western blot densitometry.

### COHb measurement

To evaluate the release of carbon monoxide (CO) from the nanoparticles, we measured carboxyhemoglobin (COHb) levels using a specific ELISA assay. COHb forms when CO binds to hemoglobin, serving as a reliable indicator of intracellular CO presence. We selected human chronic myeloid leukemia K562 cells (DSMZ, Cat# ACC10), induced to pro-erythrocyte differentiation by imatinib treatment, as these cells act as erythroid progenitors capable of producing hemoglobin. This model provides a relevant cellular system for detecting CO release and subsequent hemoglobin carboxylation within a biologically meaningful context. The COHbELISA kit (Size 96 Wells, MBS7254040) was used according to the manufacturer’s instructions on lysates from these cells, allowing sensitive and specific quantification of CO-bound hemoglobin and thereby enabling assessment of CO delivery from the nanoparticles in a cellular environment mimicking erythroid differentiation.

### Nitric oxide (NO) assay

To measure the production of NO, a critical signaling molecule in angiogenesis that promotes vasodilation, tube formation, and cell migration, we performed the Griess assay. NO concentration was measured in the culture medium of ECFCs cells using the Griess reaction. Namely, NO production was measured in cultures after overnight treatment with AuNPs_air_. Briefly, 100 µL of cell culture medium from ECFCs were mixed with an equal volume of Griess reagent (1% sulfanilamide, 0.1% N-1-naphthalenediamine dihydrochloride, and 2.5% H_3_PO_4_) and transferred to 96-well plates. Plates were incubated at room temperature for 10 min. Then the absorbance was measured at 540 nm in a microplate reader (BioTek, Winooski, VT, USA). The amount of nitrite in the media was calculated from sodium nitrite (NaNO_2_) standard curve. Results were normalized to protein concentration.

DAF-FM DA (4-amino-5-methylamino-2’,7’-difluorofluorescein diacetate) is a fluorescent probe for detecting NO in cells and tissues. The compound is non-fluorescent until it reacts with NO-derived species, resulting in a fluorescent triazole derivative. Briefly, ECFC were treated with AuNPs_air_ and CORM-2 overnight, then 5µM of DAF-FM DA (Sigma) was used to detect levels of NO. After 30’ incubation, cells were washed and incubated for 15’ with PBS (EuroClone), to obtain complete esterification of the probe, before detaching cells with accutase (EuroClone) and performing flow cytometry.

### Calcium flux detection

Calcium flux evaluation was performed with Fluo 4 AM (MedChem Express). Briefly, 1 × 10^5^ ECFC were seeded in a 6 multiwell, then 1µM of Fluo4Am was added to cell culture medium together with AuNPs_air_ or CORM-2 for 1 h. After incubation, cells were washed with PBS (EuroClone), detached with 1X Trypsin (without EDTA) and analyzed with flow cytometry.

### Immunofluorescence analysis on direct ECFC-MSC two-dimensional co-culture

For the ECFC-MSC co-culture, cells were mixed at a ratio of ECFC: MSC = 1:5 and grown on coverslips in complete EGM-2 + 10% FBS and DMEM + 20% FBS at a ratio of 1:5. Before co-culturing, ECFCs + AuNPs_air_ were incubated with culture medium containing suspensions of AuNPs at a concentration of 15 µg/mL for 24 h and then mixed with MSC. After 96 h cells were fixed with 4% paraformaldehyde and permeabilized with 0.1% Triton X100 according to routine immunocytochemistry methods. The anti-human primary antibodies used were: anti-CD31/PECAM-1 (Rabbit polyclonal 1:100, Novus) and anti-fibronectin (Mouse monoclonal 1:3000, Sigma-Merck) followed by fluorophore-conjugated secondary Abs: goat anti-rabbit Alexa Fluor 555 (Invitrogen) and goat anti-mouse Alexa Fluor 488 (Invitrogen) both diluted 1:2000. Nuclei were stained with the fluorescent DAPI (Sigma Merck) (10 µg/ml) for 15 min at RT. Sample images were acquired using TCS SP8 microscope (Leica Microsystems) with LAS-AF image acquisition software.

### RNA extraction, quantitative PCR

Total RNA was prepared using Tri Reagent (Sigma-Aldrich, Saint Louis, Missouri, USA), agarose gel checked for integrity, and reverse transcribed with cDNA synthesis kit (BioRad, Milano, Italy) according to the manufacturer’s instructions. Selected genes were evaluated by quantitative real-time PCR using SsoAdvanced Universal Green Mix (BioRad, Milano, Italy) with 7500 Fast Real Time PCR System (Applied Biosystems, Waltham, Massachusetts, USA). For real-time PCR, fold change was determined by the comparative Ct method using 18 s as normalization gene. Amplification was performed with the default PCR setting: 40 cycles of 95 °C for 10 s and 60 °C for 30 s using SYBR Green-based detection. Primer sequences (IDT, TemaRicerca, Bologna, Italy) were as follows: 18S-rRNA: sense, 5′-CCAGTAAGTGCGGGTCATAAG-3′; antisense, 5′- GCCTCACATAACCATCCAATC-3′; uPAR: sense, 5′-GCCCAATCCTGGAGCTTGA-3; antisense, 5′-TCCCCTTGCAGCTGTA-ACACT-3′; IL-8: sense, 5’-ATGACTTCCAAGCT-GGCCG-3’; antisense, 5’-TCTCAGCCCTCTTCAAAAACTT-3’; VEGF: sense, 5’-TTGCCTTGCTGCTCTACCTCCA-3’; antisense, 5’ GATGGCAGTAGCTGCGCTGATA-3’.

### In vitro capillary morphogenesis

In vitro capillary morphogenesis assay is essential for studying angiogenesis as it mimics the key steps of blood vessel formation, including endothelial cell migration, alignment, and tube formation. This method allows direct observation and quantification of the ability of endothelial cells, such as ECFCs, to form capillary-like networks under controlled conditions. In vitro capillary morphogenesis was performed in tissue culture wells coated with Matrigel (BD Biosciences). ECFCs were plated (18 × 10^3^/well) in EGM-2 medium, supplemented with 2% FBS and incubated at 37 °C- 5% CO_2_ pictures were acquired at regular intervals at EVOS optical microscope (Thermo Fisher Scientific, Monza, Italy). The Angiogenesis Analyzer tool of ImageJ software19 provided the statistical analysis for each experimental condition tested. “Nodes” are identified as pixels that had at least three neighbors, corresponding to a bifurcation. “Junctions” are elements composed of several nodes. “Segments” are elements delimited by two junctions. “Meshes” are the polygon structures reinforced with more than one layer of cells in their walls and has also been referred to by other authors as a “Honeycomb formation”.

### In vitro permeability assay

The in vitro permeability assay is used to assess how endothelial cells regulate barrier function during new blood vessel formation. Angiogenic processes involve dynamic remodeling of endothelial junctions, which modulate permeability to allow nutrient exchange and cell migration. Millicell Cell Culture Inserts (Merck Life Sciences S.r.l., Milan, Italy) were placed onto 24-weel plates and polycarbonate filters coated with 0.25 µg/µL Matrigel. ECFCs (1 × 10^5^) were cultured to confluency and treated overnight with AuNPs. Then, albumin–fluorescein isothiocyanate conjugate (BSA-FITC, Merk Life Sciences, Milan, Italy) was added to ECFCs monolayer into the luminal chamber and then the apparatus was placed in a CO_2_ incubator for 60 min. After incubation, the fluorescence of FITC-BSA cleared into the abluminal chamber was transferred to a 96-well plate, and read on the Fluoroskan Ascent FL fluorescent plate reader (Thermo Fisher Scientific, Milan, Italy) at 485 nm (excitation) and 535 nm (emission).

### Enzyme-linked immunosorbent assay (ELISA)

IL-8 concentration was measured in 100 µL of culture medium from ECFCs treated either with VEGF or AuNPs by human uncoated ELISA kits (Thermo Fisher Scientific, Milan, Italy) according to manufacturer’s instructions. IL-1α, IL-1β, IL-6, GM-CSF and MCAF concentrations were measured in 100 µL of culture medium from ECFCs treated for 24 h with AuNP_arg_, AuNP_air_, AuNPs_CO2_, CORM-2 and AuNPs_ch_ by multiplex human cytokine ELISA kits (Anogen, Ontario, Canada) according to manufacturer’s instructions.

For both ELISA kits described, the absorbance was measured at 450 nm at a microplate reader (BioTek, Winooski, VT, USA). The amount of each cytokine in the media was interpolated within the standard curves (GraphPad Prism 7 software). The results were normalized to the number of cells.

### Invasion assay with Boyden chambers

Invasion was studied in Boyden chambers in which the upper and lower wells were separated by 8 μm–pore size polycarbonate filters coated with Matrigel (BD Biosciences). ECFCs (25 × 10^3^ cells/well) were placed in the upper well of the Boyden chamber, and invasion was performed at 37 °C in 5% CO_2_ for 6 h, a time consistent with the average speeds of cell migration under mesenchymal conditions. The filters were removed and fixed in methanol. Non-invading cells on the upper surface of the filter were removed with a cotton swab, while invasive cells adherent on the lower filter surface were stained and counted using a light microscope (40x magnification) in 10 random fields/filter. Mobilization was measured by counting the number of cells moving across the filter. Each experiment was performed in triplicate and results were expressed as the absolute number of migrated cells ± SD or as % of control.

### In vivo matrigel plug assay

The in vivo Matrigel plug assay is a well-established model for evaluating angiogenesis in a physiologically relevant environment. By implanting a Matrigel matrix subcutaneously in animals, this assay allows for the assessment of new blood vessel formation in response to pro-angiogenic stimuli or treatments.

All procedures involving animals were performed in accordance with the ethical standards and according to the Declaration of Helsinki and to national guidelines approved by the ethical committee of Animal Welfare Office of Italian Health Ministry (aut. No. 326/2022-PR) and conformed to the legal mandates and Italian guidelines for the care and maintenance of laboratory animals.

Five groups of 8 and 10 four-week-old male AthymicNude-Foxn1nu mice #408,761 (three for each experimental condition) were purchased from ENVIGO. To study the ECFCs angiogenesis in vivo, the Matrigel containing ECFCs cell suspension (1 × 10^6^cells) was carefully injected subcutaneously into both flanks of mice using a cold syringe. VEGFA (10 ng/ml), was added to unpolymerized Matrigel at 4 °C at a final volume of 0.6 ml. Heparin (50 U/ml) was added to each solution. As the Matrigel warms to body temperature, it polymerizes to a solid gel, which then becomes vascularized within 10 days in response to the angiogenic substance. The extent of vascularization was quantified by performing IHC on the recovered plugs. The 15 animals were subdivided as follows: (1) three controls (Matrigel + ECFCs + Vehicle); (2) three animals injected with Matrigel + ECFCs + AuNPs_air_; (3) three animals injected with Matrigel + ECFCs + AuNPs_ch_; (4) three animals injected with Matrigel + ECFCs + AuNPs_ar_; (5) three animals injected with Matrigel + ECFCs + AuNPs_CO2_. Ten days after injection animals were sacrificed by isoflurane inhalation until complete cessation of respiration was observed, according to current Animal Welfare guidelines, and plugs were harvested and processed for histological analysis. Vascularization was evaluated by sight taking a representative photograph of individual Matrigel plugs recovered at autopsy for the corresponding condition. Samples were also fixed in formalin, embedded in paraffin for histological analysis. Following deparaffinization and rehydration, formalin-fixed plugs slices were stained with hematoxylin for 3 min (Bio Optica, Italy), rinsed in running water for 15 min, and subsequently stained with eosin for 5 min. After an additional 5 min rinse in running water, the sections were mounted using a resinous medium (09-00500, Eukitt- BioOptica Milano Spa, Italy). For anti CD31 staining, slices underwent antigen retrieval by boiling in Citrate Buffer (pH 6) at 95 °C for 10 min. To inhibit tissue peroxidases activity, the slices were then treated with a 6% H₂O₂ solution for 30 min at room temperature (RT), followed by blocking in PBS containing 2% BSA for 1 h. Next, the tissue sections were incubated overnight at 4 °C with anti-CD31 rabbit primary antibody (#SAB5600061, Merck). The sections were then incubated with anti-rabbit secondary antibody conjugated to horseradish peroxidase (HRP-linked anti-rabbit IgG, #7074, Cell Signaling Technology, USA) for 1 h, after which antigen detection was performed using the SignalStain^®^ DAB Substrate Kit (#8059, Cell Signaling Technology). DAB-positive cells were subsequently analyzed and quantified using ImageJ software [77]. Moreover, morphogenetic parameters (including master segments, master junctions, number of meshes, number of isolated meshes) were collected, quantified and visualized using GraphPad Prism 8.4.3 software.

### Statistical analysis

For statistical analysis the data were analysed using GraphPad Prism6 and Origin and expressed as a mean value ± SD. Statistical analysis was performed using One way Anova.

## Supplementary Information

Supplementary Material 1.

Supplementary Material 2.

## Data Availability

No datasets were generated or analysed during the current study.
