# Supplementary material for: Sparking angiogenesis by carbon monoxide-rich gold nanoparticles obtained by pulsed laser driven CO2 reduction reaction
Source: J Nanobiotechnology. 2025 Aug 26;23:590. doi: 10.1186/s12951-025-03680-9 (PMC12382121; doi:10.1186/s12951-025-03680-9)
Supplement: Supplementary file 1 — Supplementary Material 1. [file 12951_2025_3680_MOESM1_ESM.pdf]

Supporting Information

S1. The synthesis and characterization of the AuNPs

For the synthesis of the AuNPs by PLAL at different gas-water interface, a vacuum was first applied in the ablation chamber with water and the target, for about 1 minute. After, the gas or the gaseous mixture of interest was fluxed several times in the chamber at a pressure of about 2.5 Atm. The pressure was finally regulated at 1.2 Atm, and the system was allowed to reach gas-liquid equilibrium for a time of about 5 hours before starting the PLAL process. In Table S1 we report the laser pulse parameters used for the synthesis of the different AuNPs: ϕ is the diameter of the laser spot on the target, F and E are the fluence and the energy on the surface of the target without the presence of the AuNPs. As reported in the Materials and Methods section, for AuNPs$_{air}$ we performed a simultaneous irradiation with pulses at 1064 nm and 532 nm. In this case, we express the total fluence as $F_{1064+532}$= (F_{1064}+ F_{532}), where F_{1064} = 1.4 J/cm^2. The other AuNPs were synthesized by the use of laser pulses at the unique wavelength of 532 nm.

Sample	ϕ_{532} (mm)	E_{532} (mJ)	F_{532} (J/cm^2)	ϕ_{1064} (mm)	E_{1064} (mJ)	F_{1064} (J/cm^2)	F_{total} (J/cm^2)
AuNP$_{air}$	0.42	3	2.0	0.28	4	1.4	3.4
AuNP$_{CO2}$	0.34	7	6.5	--	--	--	6.5
AuNP$_{arg}$	0.22	30	83	--	--	--	83

Table S1. Laser pulse parameters used for the synthesis of the AuNPs by PLAL at the different gas-water interfaces. ϕ, E and F represent the diameter, energy and fluence of the laser pulses on the gold target, respectively.

Table S2 shows the gold concentration, mean size and standard deviation of the statistical size distribution of the AuNPs synthesized at the different gas-water interfaces.

	AuNP$_{air}$	AuNP$_{CO2}$	AuNP$_{arg}$	AuNP$_{ch}$
**r (nm)**	1.3 ± 0.2	3.3 ± 1.2	6.3 ± 1.5	7.4 ± 1.0
**c_{Au} (ppm)**	82 ± 4	48 ± 2	52 ± 3	38 ± 2

Table S2. Gold concentration (c_{Au}) and statistical size parameters (r) of the different synthesized AuNPs.

In Fig.S1 are presented the extinction spectra of the colloidal dispersions of nanomaterials synthesized at different gas-water interfaces, applying a normalization at the wavelength of 522 nm. As pointed out by Scaffardi et al. [1], a higher value of the interband absorption corresponds to average smaller AuNPs, so that the spectra are coherent with the TEM results shown in Fig.1. We also notice

a significant broadening of the LSPR curve in the case of $AuNPs_{arg}$, which is associated with the partial agglomeration caused by the neutral pH of the water environment in absence of NaOH.

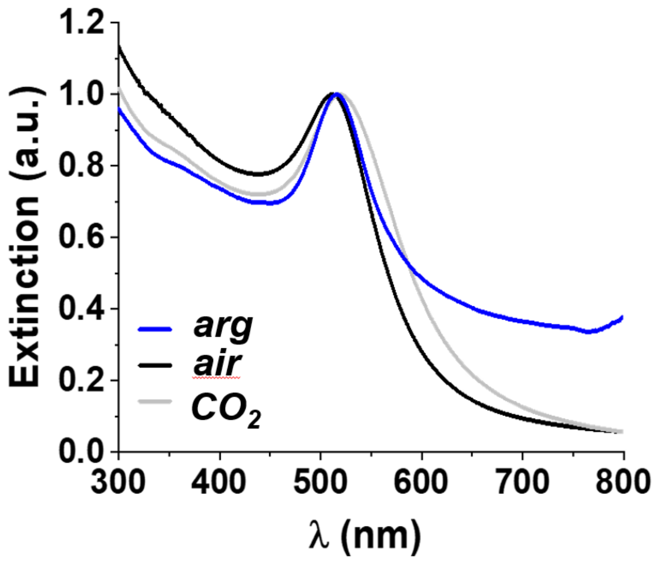

Figure S1. Extinction spectra of the AuNPs synthesized by PLAL at different gas-water interfaces.

As explained in the main text, a second-derivative spectroscopic method on the Myoglobin Soret band was used in the trials of quantification of the CO concentration in the COR-AuNPs. In particular, an adapted method proposed by Smulevich et al. was employed [2]. Myoglobin solutions (5 $\mu molL^{-1}$) were prepared in 100 mM sodium carbonate/bicarbonate buffer, adjusted to pH 9.2, this pH was selected due to the possibility of decreasing the affinity of the Mb to the O_2 and to maintain the Mb affinity to the CO [3]. For each sample, 300 µL of myoglobin solution was combined with 600 µL of buffer, 300 µL of $AuNPs_{air}$ at 82 ppm, and 100 µL of 10 mg/mL sodium dithionite solution. For samples containing L-glutathione, 100 µL of L-glutathione solution was added to the mixture to reach a final concentration of 10 $mmolL^{-1}$. The final concentration of myoglobin and $AuNPs_{air}$ were ~ 1 $\mu molL^{-1}$ and ~ 19 ppm, respectively.

All samples were fluxed in nitrogen, prepared in disposable plastic cuvettes and sealed with a layer of vaseline to prevent oxidation. UV-Vis measurements were collected in the Soret region (390-470 nm) to avoid interference from the plasmonic resonance of gold nanoparticles (COR-AuNPs). Data were acquired using a UV-Vis electronic spectrophotometer (Lambda 950, PerkinElmer, USA). In Fig.S2, we report the extinction spectra and its second derivative of myoglobin treated with sodium dithionite and/or L-glutathione mixed with the $AuNPs_{air}$.

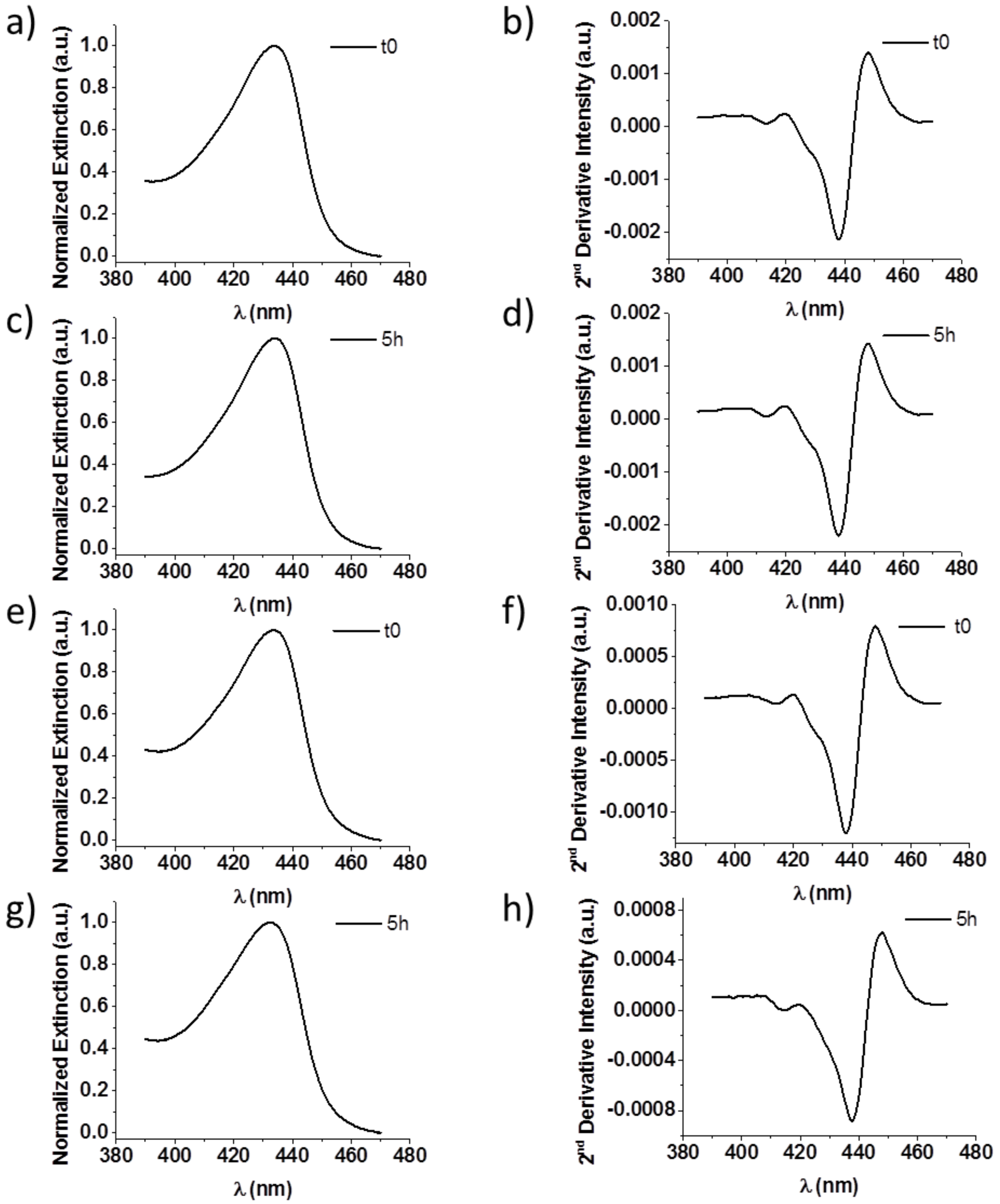

Figure S2. Spectroscopic measurements of myoglobin mixed with the $AuNPs_{air}$ at t = 0 s and t = 5h treated with sodium dithionite (a - d) or with sodium dithionite and L-glutathione (e - f). (a,c,e,f) Normalized UV-Vis spectra. (b,d,f,h) 2nd derivative of the UV-Vis spectra.

As it is visible from the spectra, the spectral position of the Soret band and its second derivative does not change along 5 hours. The bands are centered at 433 nm and 437 nm, respectively, meaning that deoxy-myoglobin remains stable without a detectable conversion to carboxy-myoglobin.

We consider two possible reasons for this particular behavior: i) the concentration of CO in the measured samples was too low to be detected by spectroscopic measurements; ii) the release of CO from the gold surface cannot be obtained by ligand-exchange with L-glutathione.

Considering the hypothesis i), a possible solution would be to use AuNPs at higher concentration, but this method is not practicable since the UV-Vis spectrometer rapidly reaches saturation with an optical density of about 2. Hence, the 20 ppm concentration of AuNPs is practically the highest concentration that can be used with a classical spectrophotometric method. Since the second-derivative method has a resolution of about 10% [[4]], if L-glutathione was able to provoke the release of CO, we should conclude that the CO concentration of the $AuNPs_{air}$ is less than 100 $nmolL^{-1}$.

The second possible explication consists in the hypothesis ii). Since, as reported in the main text, CO release is detected intracellularly, we may assume that the release can be triggered by the different possible thiol containing biomolecules in cells but not L-glutathione, or even by the so called exofacial thiols present in the cell surfaces [5].

S2. Biological activity of the AuNPs

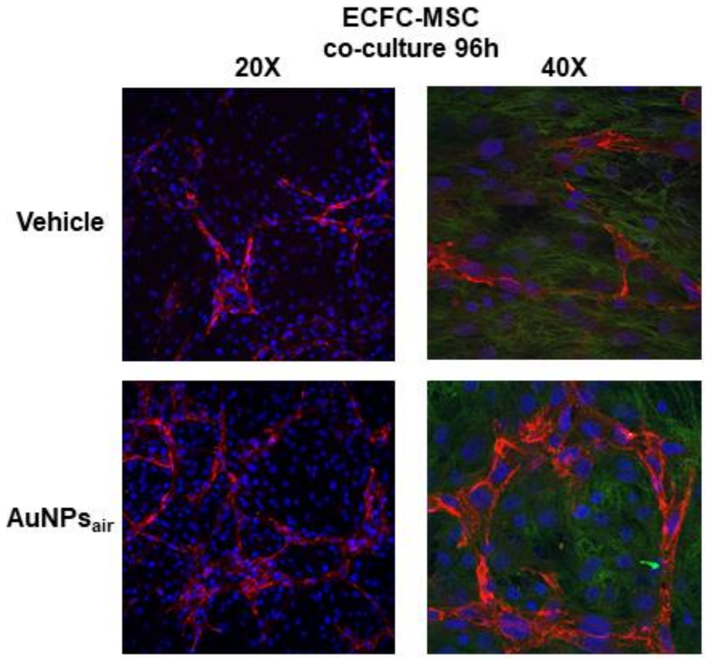

Figure S3. Immunofluorescence analysis on direct ECFC-MSC two-dimensional co-culture. ECFC and MSC were grown for 96h in a direct co-culture, plated on coverslips in complete EGM-2 + 10% FBS and DMEM + 20% FBS at an ECFC:MSC ratio of 1:5. Before co-culturing, ECFCs+$AuNPs_{air}$ were incubated with culture medium containing suspensions of AuNPs at a concentration of 15 µg/mL for 24h and then mixed with MSC. After 96h co-cultured cells were washed, fixed with 4% PFA, and stained with anti-CD31 Ab (red) and anti-fibronectin (green). The nuclei were counterstained

with DAPI (blue). Sample images (20X and 40X) were acquired using TCS SP8 microscope (Leica Microsystems) with LAS-AF image acquisition software.

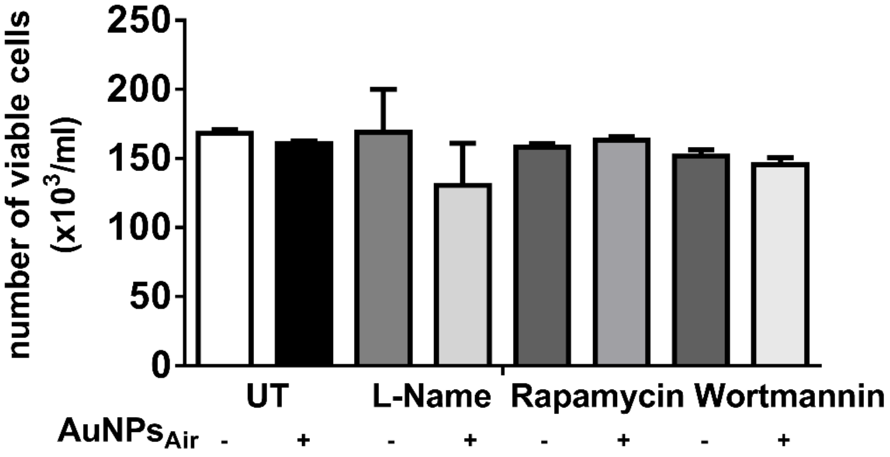

Figure S4. ECFC viability performed by trypan blue assay in control conditions (vehicle) and after treatment with AuNPs$_{air}$, in presence or absence of kinase inhibitors: L-name, Rapamycin and Wortmannin. Viable and non-viable cells (trypan blue positive) were counted separately using a dual-chamber hemocytometer and a light microscope.

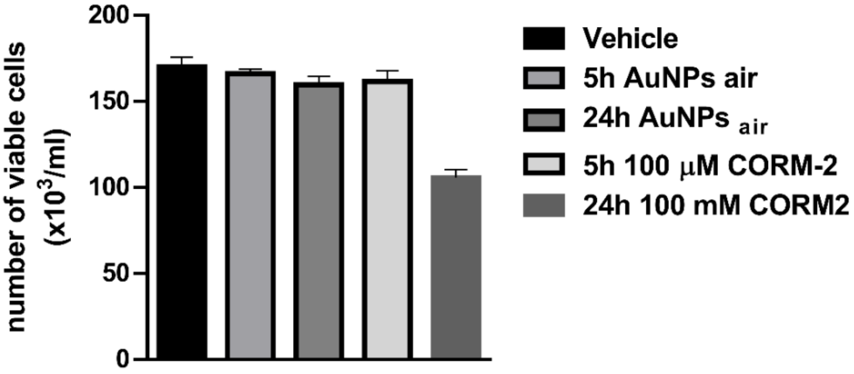

Figure S5. ECFC viability performed by trypan blue assay in control conditions (vehicle) and after treatment to either 100μM CORM-2 or 15μg/ml AuNPs$_{air}$ at specified time points: 5h and 24h. Viable and non-viable cells (trypan blue positive) were counted separately using a dual-chamber hemocytometer and a light microscope.

References

(1) Scaffardi, L. B.; Pellegri, N.; De Sanctis, O.; Tocho, J. O. Sizing Gold Nanoparticles by Optical Extinction Spectroscopy. *Nanotechnology* 2005, *16* (1), 158–163. https://doi.org/10.1088/0957-4484/16/1/030.

(2) Smulevich, G.; Droghetti, E.; Focardi, C.; Coletta, M.; Ciaccio, C.; Nocentini, M. A Rapid Spectroscopic Method to Detect the Fraudulent Treatment of Tuna Fish with Carbon Monoxide. *Food Chem* 2007, *101* (3), 1071–1077. https://doi.org/10.1016/j.foodchem.2006.03.006.

(3) Shibata, T.; Nagao, S.; Fukaya, M.; Tai, H.; Nagatomo, S.; Morihashi, K.; Matsuo, T.; Hirota, S.; Suzuki, A.; Imai, K.; Yamamoto, Y. Effect of Heme Modification on Oxygen Affinity of Myoglobin and Equilibrium of the Acid-Alkaline Transition in Metmyoglobin. *J Am Chem Soc* 2010, *132* (17), 6091–6098. https://doi.org/10.1021/ja909891q.

(4) Parks, J.; Worth, H. G. J. *Carboxyhemoglobin Determination by Second-Derivative Spectroscopy*; 1985; Vol. 31. https://academic.oup.com/clinchem/article/31/2/279/5651633.

(5) Torres, A. G.; Gait, M. J. Exploiting Cell Surface Thiols to Enhance Cellular Uptake. *Trends in Biotechnology*. Elsevier Ltd 2012, pp 185–190. https://doi.org/10.1016/j.tibtech.2011.12.002.
