# Supplementary material for: Sparking angiogenesis by carbon monoxide-rich gold nanoparticles obtained by pulsed laser driven CO2 reduction reaction
Source: J Nanobiotechnology. 2025 Aug 26;23:590. doi: 10.1186/s12951-025-03680-9 (PMC12382121; doi:10.1186/s12951-025-03680-9)
Supplement: Supplementary file 2 — Supplementary Material 2. [file 12951_2025_3680_MOESM2_ESM.docx]

**Sparking Angiogenesis by Carbon Monoxide-Rich Gold Nanoparticles obtained by Pulsed Laser Driven CO_2_ Reduction Reaction**

**How Laser Synthesized Carbon Monoxide-Rich Gold Nanoparticles Spark Angiogenesis**

Anastasia Chillà^1^*, Cecilia Anceschi^1^*, Francesca Scavone^1^, Serena Martinelli^2^, Jessica Ruzzolini^1^, Elena Frediani^1^, Francesca Margheri^1^, Tahir Tahir^3^ , Guilherme C. Concas^3^, Mariana Gisbert^3^, Marco Cremona^3^, Fernando Freire^3^, Ricardo Q. Aucélio^4^, Tatiana Saint Pierre^4^, André L. Rossi^5^, Mirko Severi^6^, Rita Traversi^6^, Daniele Bani^2^, Daniele Guasti^2^, Nicola Daldosso^7^, Mario Del Rosso^1^, Gabriella Fibbi^1^, Celso SantAnna^8^, Tommaso Del Rosso^3#,^ Anna Laurenzana^1#^

**Corresponding authors:** [tommaso@puc-rio.br](mailto:tommaso@puc-rio.br); anna.laurenzana@unifi.it

**Keywords**: carbon monoxide, gold nanoparticles, pulsed laser ablation in water, endothelial colony forming cells, capillary morphogenesis, histones acetylation; matrigel sponges; angiogenesis.

*These authors contributed equally

# These authors contributed equally

^1^ University of Florence; Department of Experimental and Clinical Biomedical Sciences,Viale Morgagni 50, 50134, Florence, Italy

^2^ Department of Experimental and Clinical Medicine University of Florence Florence 50134 Italy, Viale Pieraccini 6 50134 Florence

^3^ Pontifical Catholic University of Rio de Janeiro, Department of Physics, Rua Marquês de São Vicente 225, 22451-900 Gávea, Rio de Janeiro, Brazil

^4^ Pontifical Catholic University of Rio de Janeiro, Department of Chemistry, Rua Marquês de São Vicente 225, 22451- 900 Gávea, Rio de Janeiro, Brazil

^5^ Centro Brasileiro de Pesquisas Físicas (CBPF), R. Dr. Xavier Sigaud 150, 22290-180 Urca, Rio de Janeiro, Brazil

^2^ Department of Experimental and Clinical Medicine University of Florence Florence 50134 Italy, Viale Pieraccini 6 50134 Florence

^6^ Department of Chemistry "Ugo Schiff" University of Florence Sesto Fiorentino 50019 Italy.

^7^ University of Verona, Department of Engineering for Innovation Medicine, Strada le Grazie 15, 37134 Verona, Italy

^8^ National Institute of Metrology Quality and Technology (Inmetro), Av. Nossa Senhor das Graças 50, 25250-020

Duque de Caxias (RJ), Brazil

**Supporting Information**

**S1. The synthesis and characterization of the AuNPs**

For the synthesis of the AuNPs by PLAL at different gas-water interface, a vacuum was first applied in the ablation chamber with water and the target, for about 1 minute. After, the gas or the gaseous mixture of interest was fluxed several times in the chamber at a pressure of about 2.5 Atm. The pressure was finally regulated at 1.2 Atm, and the system was allowed to reach gas-liquid equilibrium for a time of about 5 hours before starting the PLAL process. In Table S1 we report the laser pulse parameters used for the synthesis of the different AuNPs: *φ* is the diameter of the laser spot on the target, *F* and *E* are the fluence and the energy on the surface of the target without the presence of the AuNPs. As reported in the Materials and Methods section, for AuNPs_air_ we performed a simultaneous irradiation with pulses at 1064 nm and 532 nm. In this case, we express the total fluence as *F*_1064+532_= (*F*_1064_+ *F_532_*), where *F*_1064_ = 1.4 J/cm^2^. The other AuNPs were synthesized by the use of laser pulses at the unique wavelength of 532 nm.

**
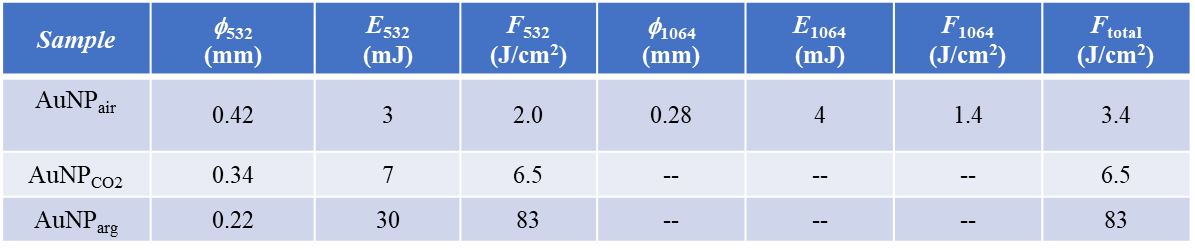
**

**Table S1.** Laser pulse parameters used for the synthesis of the AuNPs by PLAL at the different gas-water interfaces. *φ*, *E* and *F* represent the diameter, energy and fluence of the laser pulses on the gold target, respectively.

Table S2 shows the gold concentration, mean size with standard deviation of the statistical size distribution, and ζ-potential of the AuNPs synthesized at the different gas-water interfaces. In the last case, the potential was measured before (ζ_b_) and after (ζ_a_) the addition of the amphiphilic block copolymer Pluronic-F127 (PF127) in water, and also after the dilution of the colloidal dispersion with PF127 in DMEM with 10% FBS (ζ_DMEM_). In the last raw is also reported the pH of the colloidal dispersion of AuNPs immediately after the synthesis in water. The pH of DMEM with 10% FBS was 7.4.


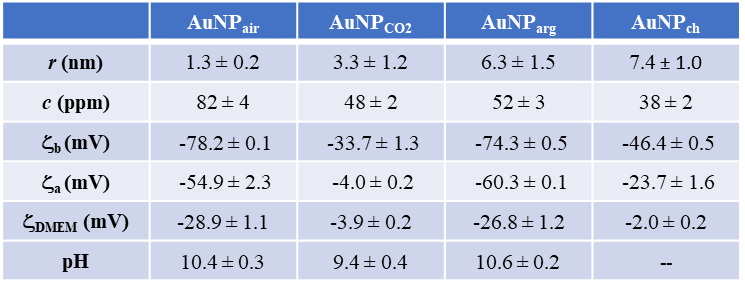


**Table S2.** Gold concentration (*c*), statistical size parameters (*r*), and ζ-potential of the different AuNPs. The ζ-potential was measured in water before (ζ_b_) and after (ζ_a_) the addition of the copolymer PF127, and also in DMEM (10% FBS) after the addition of PF127 (ζ_DMEM_).

In Fig.S1 are presented the extinction spectra of the colloidal dispersions of nanomaterials synthesized at different gas-water interfaces, applying a normalization at the wavelength of 522 nm. As pointed out by Scaffardi et al. [1], a higher value of the interband absorption corresponds to average smaller AuNPs, so that the spectra are coherent with the TEM results shown in Fig.1. We also notice a significant broadening of the LSPR curve in the case of AuNPs_arg_, which is associated with the partial agglomeration caused by the neutral pH of the water environment in absence of NaOH.


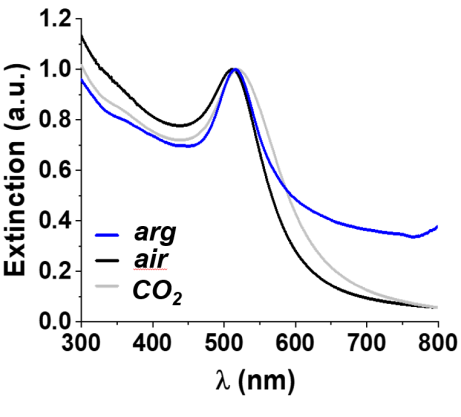


**Figure S1.** Extinction spectra of the AuNPs synthesized by PLAL at different gas-water interfaces.

As explained in the main text, a second-derivative spectroscopic method on the Myoglobin Soret band was used in the trials of quantification of the CO concentration in the COR-AuNPs. In particular, an adapted method proposed by Smulevich et al. was employed [2]. Myoglobin solutions (5 μmolL^-1^) were prepared in 100 mM sodium carbonate/bicarbonate buffer, adjusted to pH 9.2, this pH was selected due to the possibility of decreasing the affinity of the Mb to the O_2_ and to maintain the Mb affinity to the CO [3]. For each sample, 300 µL of myoglobin solution was combined with 600 µL of buffer, 300 µL of AuNPs_air_ at 82 ppm, and 100 µL of 10 mg/mL sodium dithionite solution. For samples containing L-glutathione, 100 µL of L-glutathione solution was added to the mixture to reach a final concentration of 10 mmolL^-1^ For the samples containing DMEM, 100 mmolL^-1^ of the culture media was added to the mixture to reach a final concentration of 10 mmolL^-1^.The final concentration of myoglobin and AuNPs_air_ were ~ 1 μmolL^-1^ and ~ 19 ppm, respectively.

All samples were fluxed in nitrogen, prepared in disposable plastic cuvettes and sealed with a layer of vaseline to prevent oxidation. UV-Vis measurements were collected in the Soret region (390-470 nm) to avoid interference from the plasmonic resonance of gold nanoparticles (COR-AuNPs). Data were acquired using a UV-Vis electronic spectrophotometer (Lambda 950, PerkinElmer, USA). In Fig.S2, we report the extinction spectra and its second derivative of myoglobin treated with sodium dithionite and/or L-glutathione mixed with the AuNPs_air_.


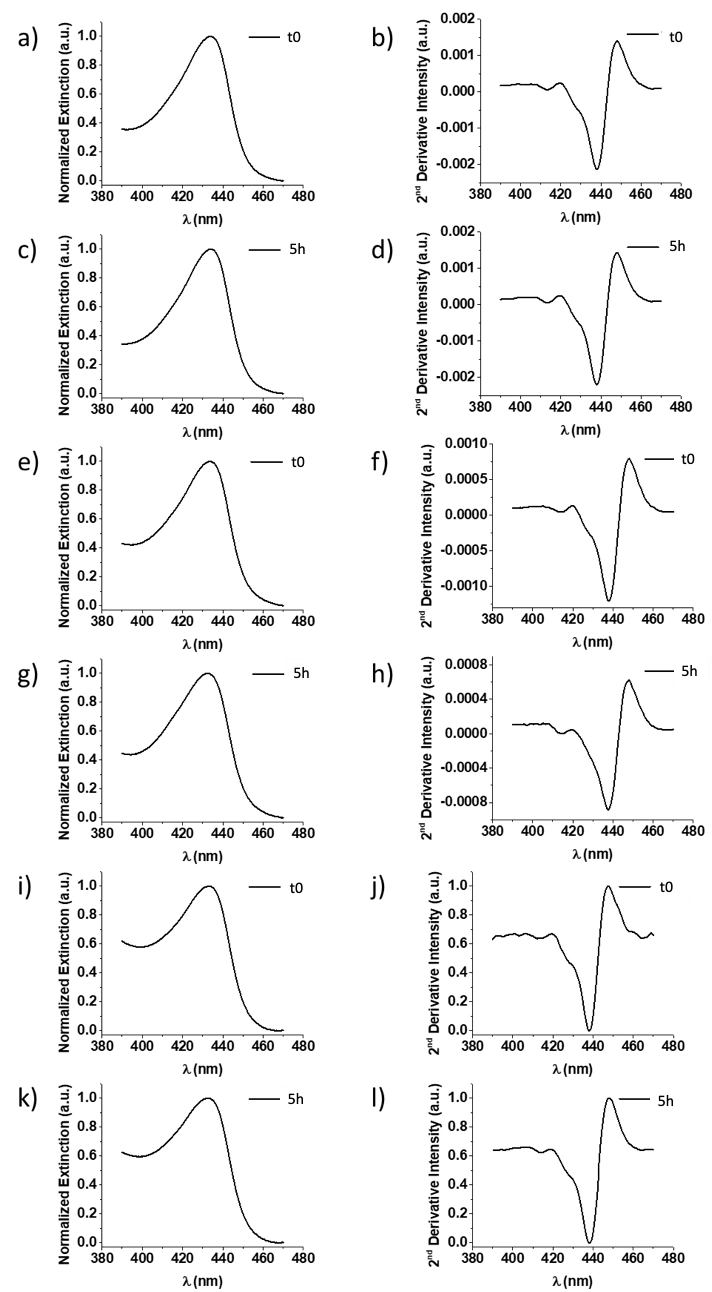


**Figure S2.** Spectroscopic measurements of myoglobin mixed with the AuNPs_air_ at t = 0 s and t = 5h treated with sodium dithionite (a - d), with sodium dithionite and L-glutathione (e - f), and with DMEM (i - l). (a,c,e,f,i,k) Normalized UV-Vis spectra. (b,d,f,h,j,l) 2^nd^ derivative of the UV-Vis spectra.

As it is visible from the spectra, the spectral position of the Soret band and its second derivative does not change for 5 hours. The bands are centered at 433 nm and 437 nm, respectively, meaning that deoxy-myoglobin remains stable without a detectable conversion to carboxy-myoglobin.

We consider two possible reasons for this behavior: i) the concentration of CO in the measured samples was too low to be detected by spectroscopic measurements; ii) the release of CO from the gold surface cannot be obtained by ligand-exchange with L-glutathione.

Considering the hypothesis i), a possible solution would be to use AuNPs at higher concentration, but this method is not practicable since the UV-Vis spectrometer rapidly reaches saturation with an optical density of about 2. Hence, the 20 ppm concentration of AuNPs is practically the highest concentration that can be used with a classical spectrophotometric method. Since the second-derivative method has a resolution of about 10% [4], if L-glutathione was able to provoke the release of CO, we should conclude that the CO concentration of the AuNPs_air_ is less than 100 nmolL^-1^.

The second possible explication consists in the hypothesis ii). Since, as reported in the main text, CO release is detected intracellularly, we may assume that the release can be triggered by the different possible thiol containing biomolecules in cells but not L-glutathione, or even by the so called exofacial thiols present in the cell surfaces [5].


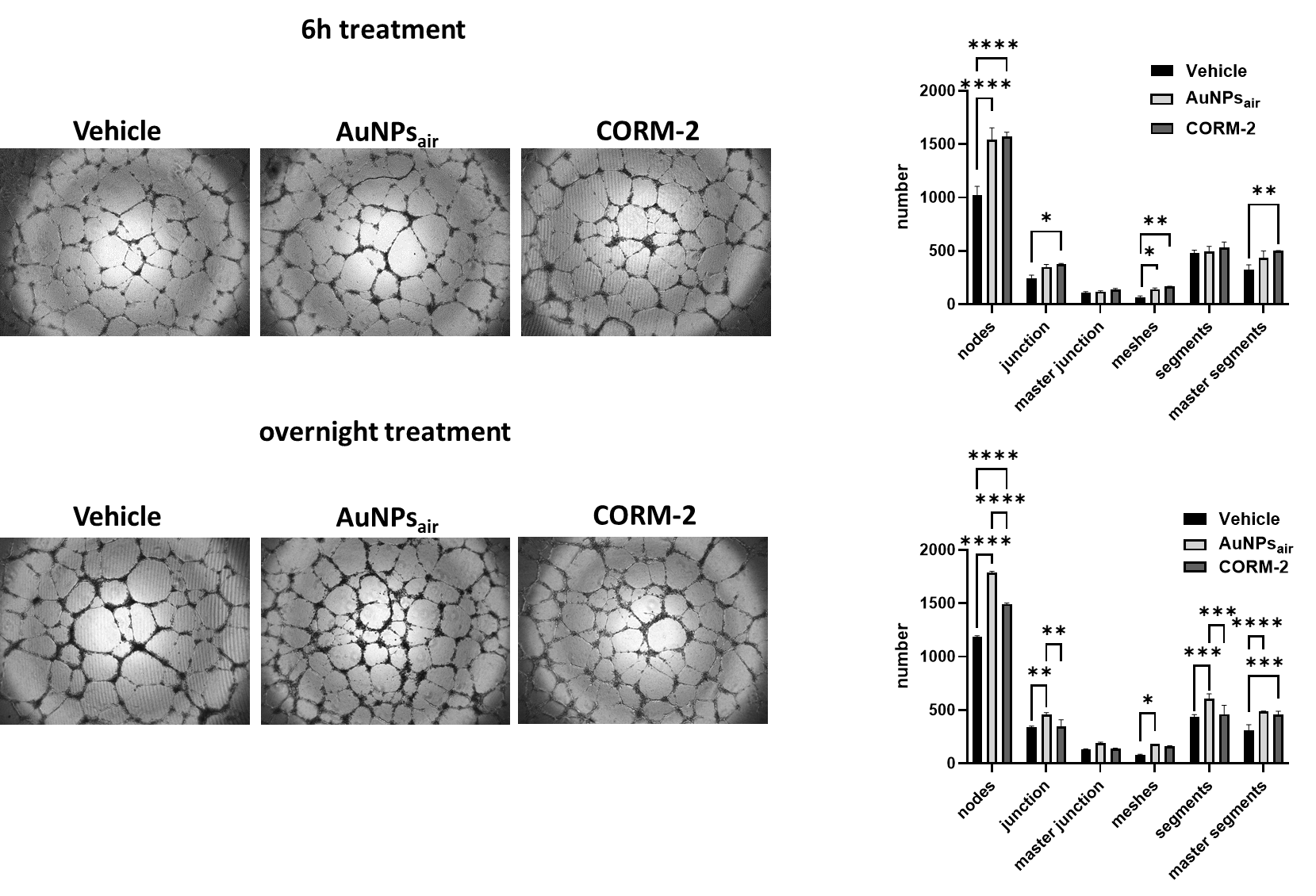


Figure S3. Optical images of EFCF after 6-hour and overnight treatment with AuNPs_air_ or CORM-2, taken 24 hours after the initiation of the capillary morphogenesis assay. A total of 18,000 cells-either vehicle-treated or treated with AuNPs_air_ or CORM-2, -were seeded on tissue culture wells coated with Matrigel. Images were captured following the formation of vessel-like structures at EVOS optical microscope. Quantification of capillary structures (on the right) was performed using the Angiogenesis Analyzer tool of ImageJ (see material and method section). Results represent the mean of three independent experiments performed in duplicate. Error bars indicate mean ± SD; p < 0.05 denotes a significant difference compared to the vehicle.


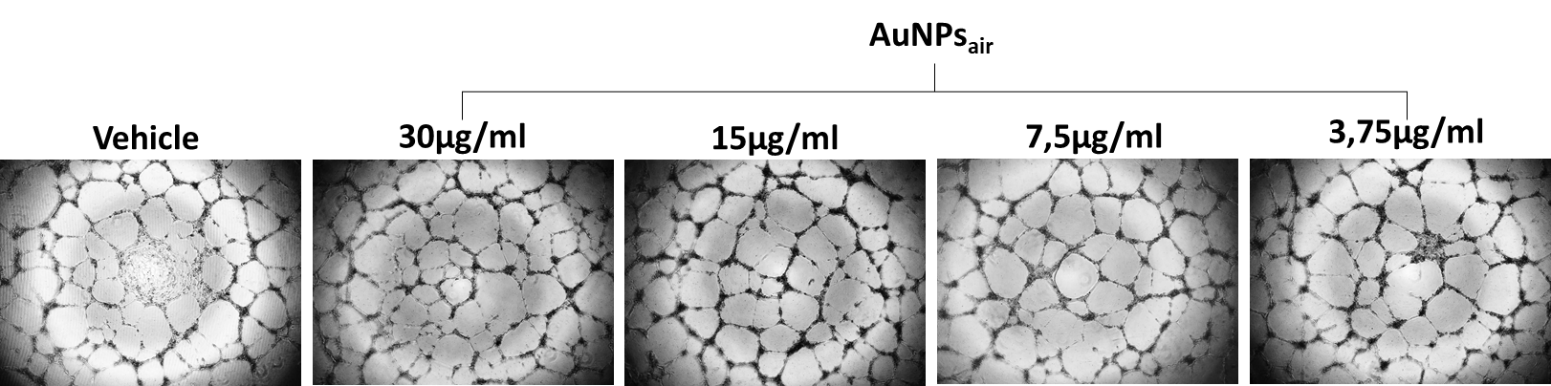


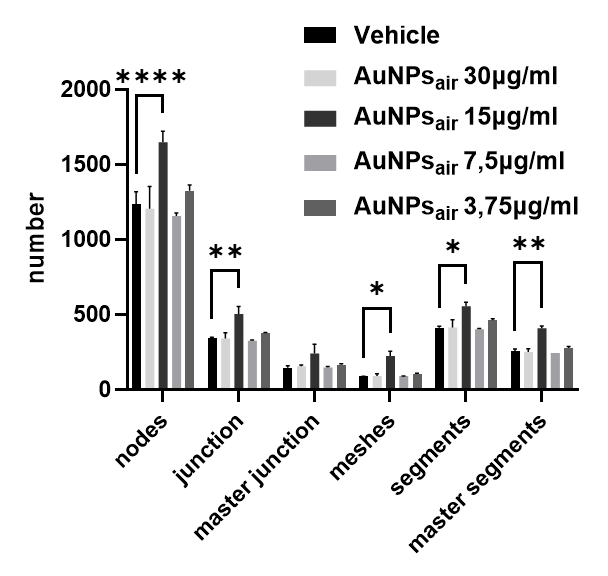


**Figure S4.** Optical images of EFCF after overnight, dose-dependent treatment with AuNPs_air_ (top panel), taken 24 hours after the initiation of the capillary morphogenesis assay. A total of 18,000 cells- either vehicle-treated or treated with AuNPs _air_ - were seeded on tissue culture wells coated with Matrigel. Images were captured following the formation of vessel-like structures at EVOS optical microscope. Quantification of capillary structures (bottom panel) was performed using the Angiogenesis Analyzer tool of ImageJ (see material and method section). Results represent the mean of three independent experiments performed in duplicate. Error bars indicate mean ± SD; p < 0.05 denotes a significant difference compared to the vehicle.


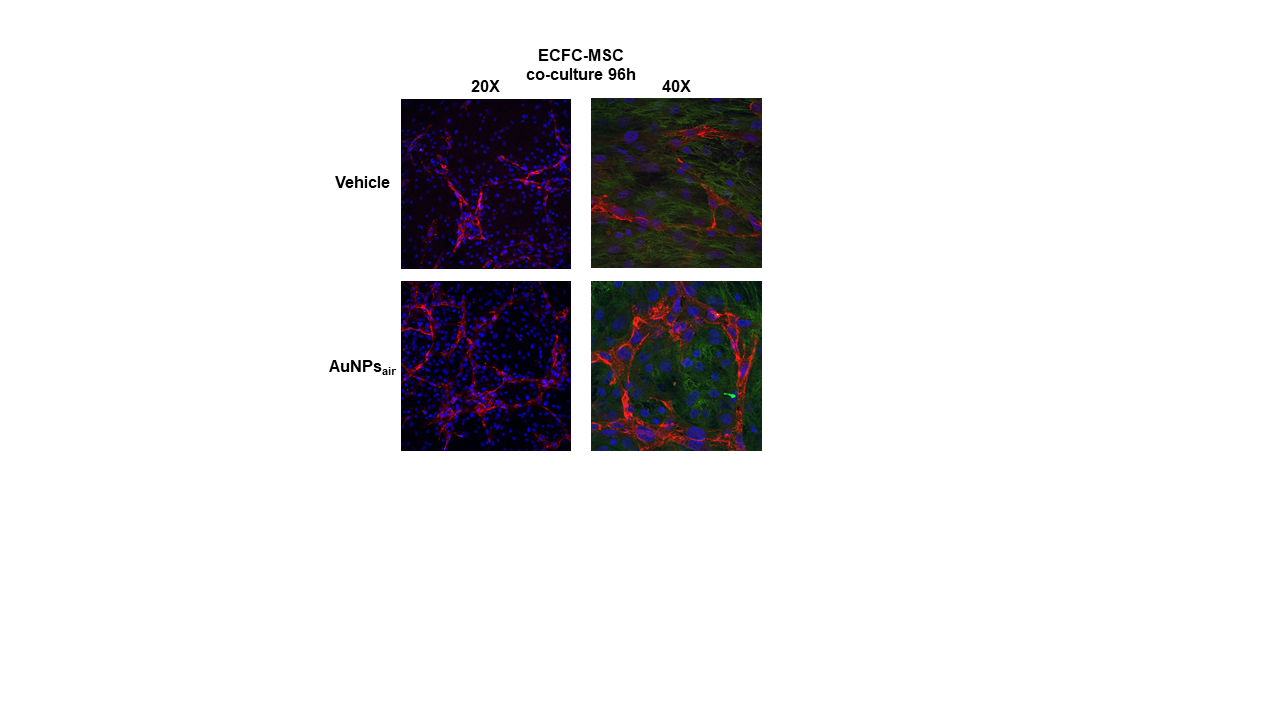


**Figure S5.** Immunofluorescence analysis on direct ECFC-MSC two-dimensional co-culture. ECFC and MSC were grown for 96h in a direct co-culture, plated on coverslips in complete EGM-2 + 10% FBS and DMEM + 20% FBS at an ECFC:MSC ratio of 1:5. Before co-culturing, ECFCs+AuNPs_air_ were incubated with culture medium containing suspensions of AuNPs at a concentration of 15 μg/mL for 24h and then mixed with MSC. After 96h co-cultured cells were washed, fixed with 4% PFA, and stained with anti-CD31 Ab (red) and anti-fibronectin (green). The nuclei were counterstained with DAPI (blue). Sample images (20X and 40X) were acquired using TCS SP8 microscope (Leica Microsystems) with LAS-AF image acquisition software.


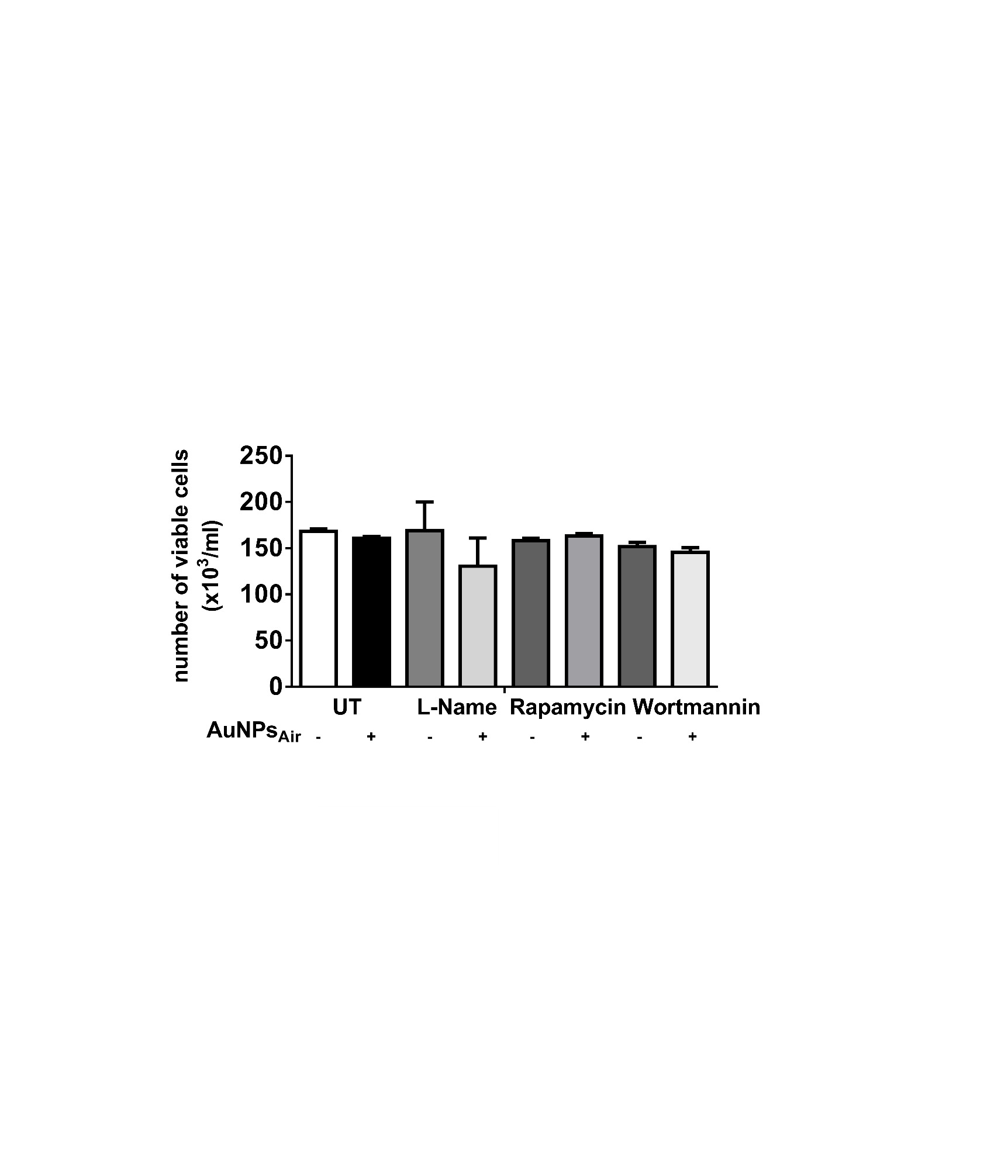


**Figure S6.** ECFC viability performed by trypan blue assay in control conditions (vehicle) and after treatment with AuNPs_air,_ in presence or absence of kinase inhibitors: L-name, Rapamycin and Wortmannin. Viable and non-viable cells (trypan blue positive) were counted separately using a dual-chamber hemocytometer and a light microscope.

**
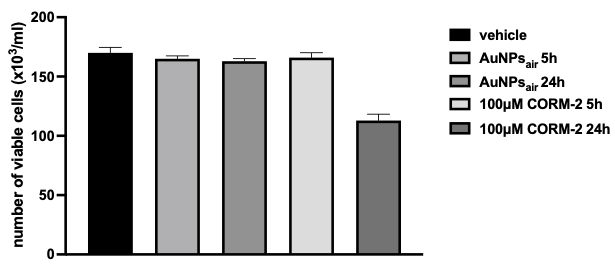
**

**Figure S7** ECFC viability performed by trypan blue assay in control conditions (vehicle) and after treatment to either 100µM CORM-2 or 15µg/ml AuNPs_air_ at specified time points: 5h and 24h. Viable and non-viable cells (trypan blue positive) were counted separately using a dual-chamber hemocytometer and a light microscope.


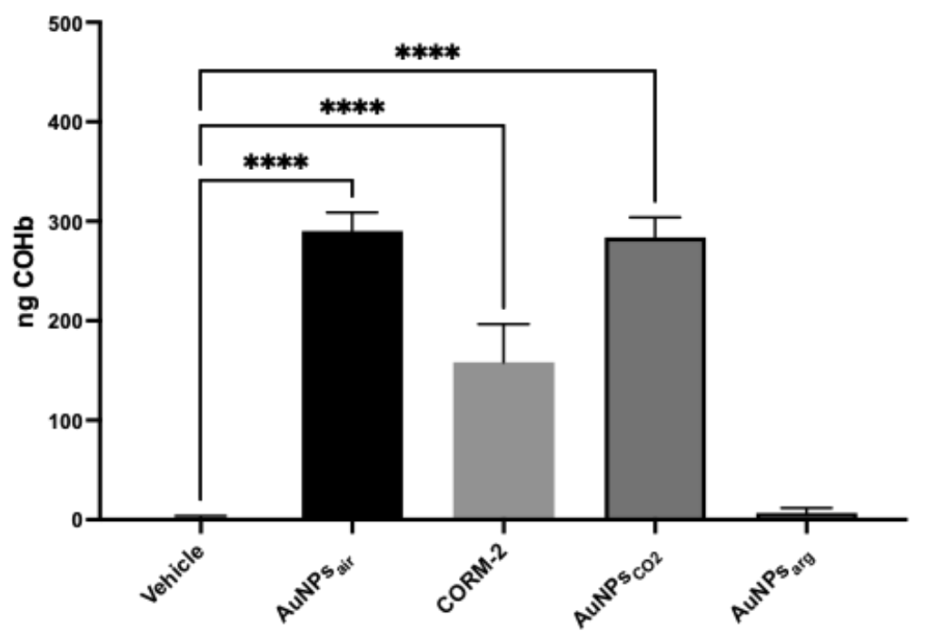


**Figure S8.** Quantification of CoHB released by K562 treated for 6h with CORM-2 AuNPs_air_, AuNPs_arg_, and AuNPs_CO2._ Results represent the mean of three independent experiments performed in duplicate. Error bars indicate mean ± SD; p < 0.05 denotes a significant difference compared to the vehicle


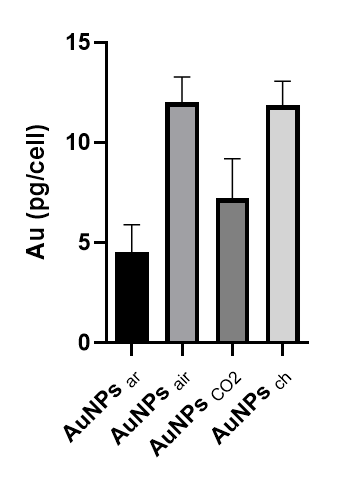


**Figure S9** ICP-AES analysis of AuNPs loaded ECFCs.


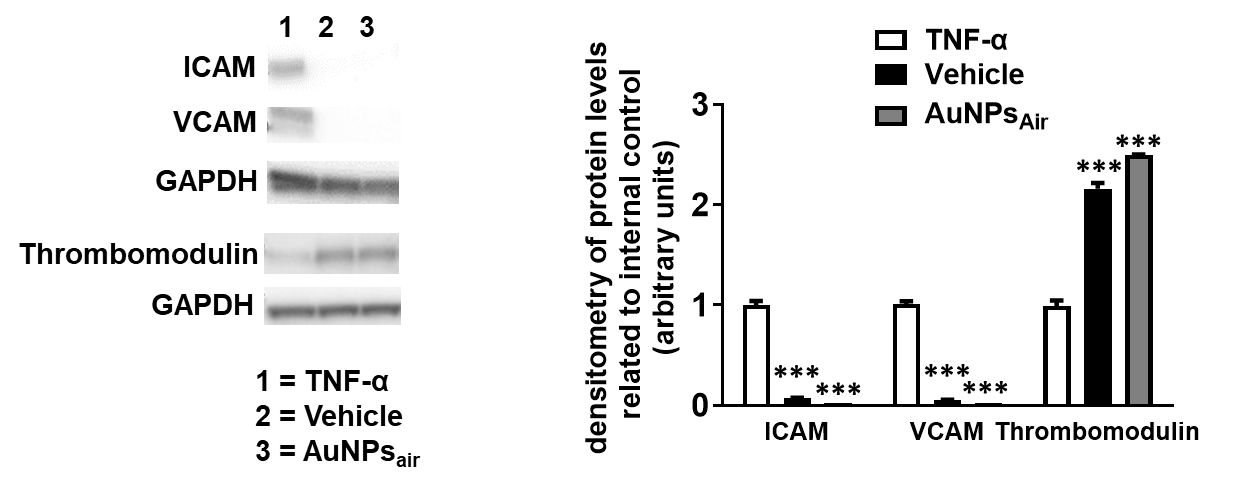


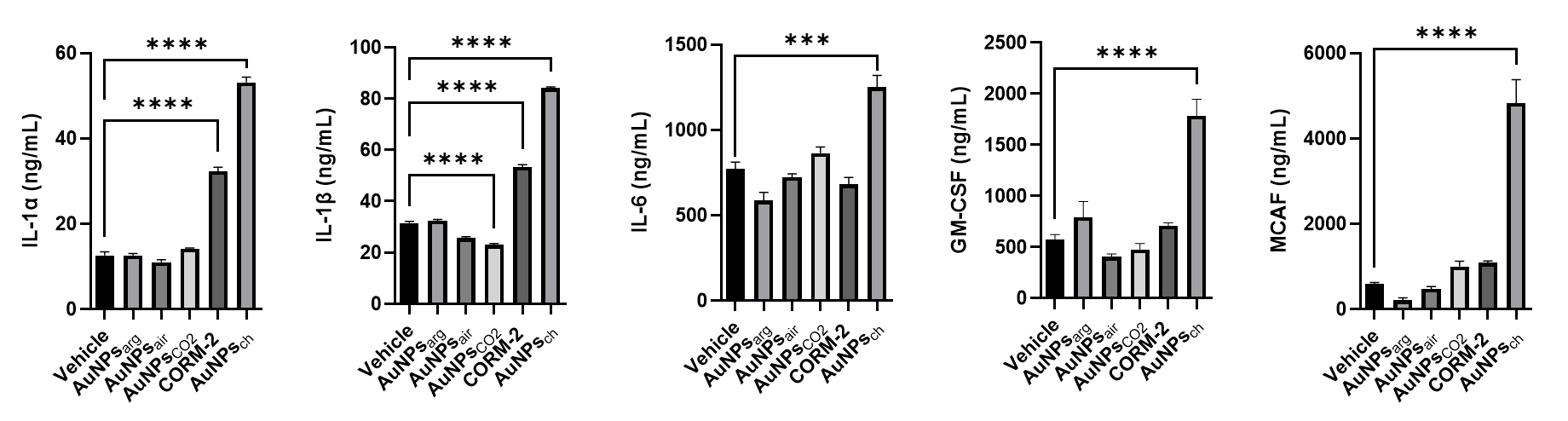


**Figure S10**. Western blot analysis of ICAM-1 and VCAM-1, two adhesion molecules involved in leukocyte–endothelial adhesion, and thrombomodulin, an endothelial cell surface receptor involved in anticoagulation, in ECFC treated with vehicle, TNF-α (positive control), or AuNPs_air_. GAPDH was used as a loading control (upper panel). Histograms in the show band densitometry quantification (upper panel). Results represent the mean of three independent experiments performed in duplicate. Error bars indicate mean ± SD; p < 0.05 denotes a significant difference compared to the vehicle. Quantification of pro-inflammatory cytokines by ELISA. Levels of IL-1α, IL-6, IL-1β, Granulocyte-Macrophage Colony-Stimulating Factor (GM-CSF), and Monocyte Chemotactic and Activating Factor (MCAF/MCP-1) were measured in culture supernatants of ECFC treated with vehicle, AuNPs_air_, AuNPs_arg_, and AuNPs_CO2_, AuNPs_ch_ using specific sandwich ELISA kits. Cytokine concentrations were calculated based on standard curves generated with recombinant proteins. with recombinant standards. Data are expressed as mean ± SD of at least three independent experiments. Statistical significance was assessed using one-way ANOVA; *p* < 0.05 was considered significant.

**References**

1. Scaffardi LB, Pellegri N, de Sanctis O, Tocho JO: **Sizing gold nanoparticles by optical extinction spectroscopy.** *Nanotechnology* 2005, **16:**158.

2. Smulevich G, Droghetti E, Focardi C, Coletta M, Ciaccio C, Nocentini M: **A rapid spectroscopic method to detect the fraudulent treatment of tuna fish with carbon monoxide.** *Food Chemistry* 2007, **101:**1071-1077.

3. Shibata T, Nagao S, Fukaya M, Tai H, Nagatomo S, Morihashi K, Matsuo T, Hirota S, Suzuki A, Imai K, Yamamoto Y: **Effect of heme modification on oxygen affinity of myoglobin and equilibrium of the acid-alkaline transition in metmyoglobin.** *J Am Chem Soc* 2010, **132:**6091-6098.

4. Parks J, Worth HG: **Carboxyhemoglobin determination by second-derivative spectroscopy.** *Clin Chem* 1985, **31:**279-281.

5. Torres AG, Gait MJ: **Exploiting cell surface thiols to enhance cellular uptake.** *Trends Biotechnol* 2012, **30:**185-190.
